# Supplementary material for: Changes of perceptions and behaviours during the phases of COVID-19 pandemic in German elderly people with neurological disorders: an observational study using telephone interviews
Source: BMJ Open. 2021 Jan 12;11(1):e045780. doi: 10.1136/bmjopen-2020-045780 (PMC7804827; doi:10.1136/bmjopen-2020-045780)
Supplement: Author's manuscript [file bmjopen-2020-045780.draft_revisions.pdf]

BMJ Open

BMJ Open is committed to open peer review. As part of this commitment we make the peer review history of every article we publish publicly available.

When an article is published we post the peer reviewers' comments and the authors' responses online. We also post the versions of the paper that were used during peer review. These are the versions that the peer review comments apply to.

The versions of the paper that follow are the versions that were submitted during the peer review process. They are not the versions of record or the final published versions. They should not be cited or distributed as the published version of this manuscript.

BMJ Open is an open access journal and the full, final, typeset and author-corrected version of record of the manuscript is available on our site with no access controls, subscription charges or pay-per-view fees (http://bmjopen.bmj.com).

If you have any questions on BMJ Open's open peer review process please email info.bmjopen@bmj.com

BMJ Open

Changes of Perceptions and Behaviours during the Phases of COVID-19 Pandemic in German Elderly People with Neurological Disorders: An Observational Study using Telephone Interviews

Journal:	*BMJ Open*
Manuscript ID	bmjopen-2020-045780
Article Type:	Original research
Date Submitted by the Author:	12-Oct-2020
Complete List of Authors:	Zipprich, Hannah; Jena University Hospital, Neurology<br>Schönenberg, Aline; Jena University Hospital, Neurology<br>Teschner, Ulrike; Jena University Hospital, Neurology<br>Prell, Tino; Jena University Hospital, Neurology
Keywords:	Neurology < INTERNAL MEDICINE, COVID-19, Anxiety disorders < PSYCHIATRY

SCHOLARONE™
Manuscripts

I, the Submitting Author has the right to grant and does grant on behalf of all authors of the Work (as defined in the below author licence), an exclusive licence and/or a non-exclusive licence for contributions from authors who are: i) UK Crown employees; ii) where BMJ has agreed a CC-BY licence shall apply, and/or iii) in accordance with the terms applicable for US Federal Government officers or employees acting as part of their official duties; on a worldwide, perpetual, irrevocable, royalty-free basis to BMJ Publishing Group Ltd ("BMJ") its licensees and where the relevant Journal is co-owned by BMJ to the co-owners of the Journal, to publish the Work in this journal and any other BMJ products and to exploit all rights, as set out in our licence.

The Submitting Author accepts and understands that any supply made under these terms is made by BMJ to the Submitting Author unless you are acting as an employee on behalf of your employer or a postgraduate student of an affiliated institution which is paying any applicable article publishing charge ("APC") for Open Access articles. Where the Submitting Author wishes to make the Work available on an Open Access basis (and intends to pay the relevant APC), the terms of reuse of such Open Access shall be governed by a Creative Commons licence – details of these licences and which Creative Commons licence will apply to this Work are set out in our licence referred to above.

Other than as permitted in any relevant BMJ Author's Self Archiving Policies, I confirm this Work has not been accepted for publication elsewhere, is not being considered for publication elsewhere and does not duplicate material already published. I confirm all authors consent to publication of this Work and authorise the granting of this licence.

For peer review only

BMJ open

Changes of Perceptions and Behaviours during the Phases of COVID-19 Pandemic in German Elderly People with Neurological Disorders: An Observational Study using Telephone Interviews

Hannah M. Zipprich, MD[1,2], Aline Schönenberg[1], Ulrike Teschner, PhD[1] and Tino Prell, MD[1,2,*]

[1] Department of Neurology, Jena University Hospital, Jena, Germany
[2] Center for Healthy Ageing, Jena University Hospital, Jena, Germany

* Correspondence: Tino.prell@med.uni-jena.de; Tel.: +49 3641 9323497

Word count (excl. title page, abstract, references, figures and tables): 1969 words

For peer review only

ABSTRACT

Objectives: Describing perceived limitations in everyday life, psychological burden and approval to easing of measures during the COVID-19 phases in elderly people with neurological disorders.

Design: Observational, prospective study

Setting: This is a monocentric study conducted at a university hospital in Germany.

Participants: Overall, 452 elderly people participated in the NeuroGerAdh study (DRKS00016774) and were interviewed by telephone between March 18 and August 30, 2020.

Results: Overall, 307 (67.9%) patients had relevant limitations in daily life due to the measures. These limitations significantly decreased during the pandemic phases. At the beginning of the pandemic, people complained about restricted social contacts and mobility, which were the most common reasons for perceived limitations in daily life. Later, at phase three, wearing a mouth–nose mask had become the main reason for perceived limitations. Higher perceived limitations were associated with younger age and earlier pandemic phases. Moreover, the perceived psychological burden decreased as the pandemic phases passed, even though the reasons for psychological burden (anxiety or fear of infection, insecurity and concerns) did not remarkably change during the phases. Higher psychological burden, Beck Depression Inventory II and Montreal Cognitive Assessment were associated with early pandemic phases. From June 16, 2020, the patients were asked whether they approve the easing of measures. Sixty-seven of 136 patients (49.3%) approved and 55 (40.4%) did not. The common reasons for disapproval were fear of increased risk of infection and irresponsible behaviour of other people.

Conclusion: While limitations in daily life decreased during the study period, anxiety remains a common psychological burden in elderly sick people, and this needs special attention. Accordingly, most people do not approve easing of measures. Special strategies are needed to cope with changing measures during the COVID-19 pandemic.

KEYWORDS: neurogeriatric; COVID-19; anxiety; easing of measures

For peer review only

ARTICLE SUMMARY

Strengths and limitations of this study

- The inclusion of underrepresented elderly sick people.
- The availability of clinical data for depression, cognitive ability and mobility for the telephone-interviewed participants.
- The monocentric study design and focus on people with neurological disorders.
- The cross-sectional design does not allow statements about causality.

For peer review only

INTRODUCTION

Severe acute respiratory syndrome coronavirus 2 (SARS-CoV-2), a novel virus causing coronavirus disease 2019 (COVID-19) infection, has resulted in a pandemic. Local and national governments have taken unprecedented measures in response to the outbreak of SARS-CoV-2-induced COVID-19, including the isolation of patients, enforcement of quarantine of all contacts, cancellation of public transportation, exit controls, travel restrictions, social contact restrictions and requirement of people to wear mouth–nose masks.[1,2]

Considering the globally and locally rising infection rates, the German federal state issued different ordinances to contain the spread of the virus. The first ordinance published in March 2020 included a lockdown with limited social contacts, maintaining a minimum distance of at least 1.5m in public and closing of service establishments and restaurants as well as teaching facilities. A maximum of two people from different households were allowed to meet (see also Thüringer Verordnung über erforderliche Maßnahmen zur Eindämmung der Ausbreitung des Coronavirus SARS CoV 2, 2020). Since April 2020, governments have been requiring people to wear mouth–nose masks in shops and public transportation. In May 2020, Germany announced relevant easing of measures against the coronavirus pandemic: resuming regular operating hours of kindergartens and schools, reopening of restaurants and allowing meetings of up to five people from different households. Since the beginning of June 2020, further easing of measures has been occurring. For example, visits to nursing homes and larger family celebrations are possible again. Thuringia, in particular, was the first federal state to lift the contact restrictions. The new standard regulations deployed since June 2020 still require people to practice social distancing and wear mouth–nose masks in shops and public transportation, but contain only recommendations and no rules for restricting social contacts (see also Thüringer Verordnung zur Neuordnung der erforderlichen Maßnahmen zur Eindämmung der Ausbreitung des Coronavirus SARS-CoV-2, 2020).

It is understandable that such drastic measures have a considerable impact on the physical and psychological well-being of the population.[3,4] Especially, the elderly population, being particularly vulnerable to severe cases of COVID-19, is at risk of increased anxiety and depression.[5] So far, however, the effect of the measures in the older population and

For peer review only

changes in perceptions when measures were being relaxed have not been sufficiently investigated. Therefore, we explored the impact of the measures during the three phases of the coronavirus pandemic in Germany: Phase 1 (March 18, 2020–May 15, 2020) with the lockdown, Phase 2 (May 16, 2020–June 05, 2020) with the first easing of measures and Phase 3 (since June 06, 2020) with further easing of measures.

METHODS

Study design and assessments

The people who were enrolled in the NeuroGerAdh study (DRKS00016774) were interviewed via telephone between March 18, 2020 and August 30, 2020. The NeuroGerAdh study is a longitudinal observational study of predictors of nonadherence to medication in patients with neurological disorders.[6] Here, elderly people who were treated at the Department of Neurology at Jena University Hospital were consecutively recruited and assessed using comprehensive geriatric assessment and an adherence questionnaire and two follow-up telephone calls to explore changes in medication after discharge from hospital. The study was approved by the local ethics committee (approval number 5290-10/17) of Jena University Hospital. All patients provided written informed consent. During the COVID-19 pandemic, the following questions were added to the interview:

1. Are or were you infected with coronavirus? Yes/no (if yes, when?)
2. Are or have you already been quarantined? Yes/no (if yes, how long?)
3. For which activities do you still leave the house/apartment?
    - Shopping
    - Visiting to the doctor
    - Walking/hiking/cycling
    - Working in the garden
    - Doing sports
    - Visiting family
    - Meeting with friends
    - Not at all, I am being cared for
4. Please indicate on a scale of 0–10 how much you are limited in daily life by the measures against COVID-19 (0 being 'no limitation of my daily life' and 10 being

For peer review only

‘strongest restrictions of my daily life’). If >0, please specify what you are limited by/what you feel is a limitation.

5. Please indicate on a scale of 0–10 how much of a psychological burden the restrictions put on you (0 being ‘not burdening at all’ and 10 being ‘very strongly burdening’).

6. Since June 16, 202,0 we additionally asked, ‘Do you approve the easing of the measures?’ Yes/no (if no, why?)

7. From June 16, 2020, the people were also asked, ‘Do you approve the easing of the measures?’ Yes/no (if no, why?)

Answers to questions 4–7 were evaluated qualitatively and categorised into specific subject areas. In addition, the following data were used for analyses: age, gender, main neurological disorder, marital status (single, divorced/widowed or married), level of education (high: German *Abitur* or university; medium: German *Realschule* or the General Certificate of Secondary Education; low: German *Hauptschule* or no school), cognitive function (Montreal Cognitive Assessment (MoCA)), depressive mood (Beck Depression Inventory II (BDI-II)), patient–physician relationship (Health Care Climate Questionnaire (HCCQ)) and Timed ‘Up & Go’ (TUG) test in seconds to assess a person’s mobility and risk of falling.

Patient and Public Involvement statement

Patients or the public were not involved in the design, or conduct, or reporting, or dissemination plans of our research.

Statistical analysis

Statistical analysis was performed using Statistical Package for the Social Sciences (version 25.0; IBM Corp., Armonk, NY, USA). The data were analysed using descriptive statistics: means, standard deviations, frequencies and percentages. No techniques were used to replace missing data. Comparisons of clinical parameters between patients from the three phases were performed using the Kruskal–Wallis and chi-square tests. Linear regression with backward selection was used to study the association between perceived limitations in daily life and perceived psychological burden with the following independent variables: age, gender, marital state (married or unmarried), education level (high, middle and low),

For peer review only

pandemic phase, BDI, HCCQ, MoCA and TUG in seconds. The significance levels for variables included the linear regression model and those removed from the model were set at 0.05 and 0.1, respectively. Before regression analyses, we excluded autocorrelation (Durbin–Watson) and multicollinearity (variance inflation factor and tolerance).

RESULTS

Among the 452 people were interviewed in this study (Table 1), 230 (50.9%) were interviewed in Phase 1, 41 (9.1%) in Phase 2 and 181 (40.0%) in Phase 3. Only two people (0.4%) had COVID-19 infection and 18 people (4%) had been in quarantine before the interview. The people who were interviewed in the three phases did not differ in terms of age ($p = 0.68$), BDI ($p = 0.16$), HCCQ ($p = 0.76$), MoCa ($p = 0.49$), TUG ($p = 0.38$), gender ($p = 0.53$), marital state ($p = 0.58$) and education level ($p = 0.69$). The raw data (including missing values) are given in the supplement.

Table 1. Characteristics of the entire cohort

		**m**	**SD**
Age		69.5	8.6
Beck Depression Inventory II		9.7	7.4
Health Care Climate Questionnaire		5.9	1.1
Montreal Cognitive Assessment		22.8	4.7
Timed Up & Go		10.6	4.5
		**n**	**%**
Sex	Female	205	45.4
	Male	247	54.6
Marital status	Single	131	29.5
	Married	313	70.5
Education level	High	153	34.5
	Middle	156	35.2
	Low	134	30.2
Diagnosis group	Movement disorders	125	27.7
	Cerebrovascular disorders	116	25.7
	Epilepsy	24	5.3
	Neuromuscular disorders	105	23.2
	Others	82	18.1

For peer review only

Overall, 307 (67.9%) people reported having relevant limitations in their daily routine. The perceived limitations in daily life caused by the measures decreased significantly during the pandemic phases (Figure 1). The slight increase in perceived limitations from Phase 2 to Phase 3 was not significant. The reasons for the perceived limitations changed during the pandemic phases. At the beginning of the pandemic, people considered restricted social contacts and mobility as the most common reasons for perceived limitations in daily life. Later, at Phase 3, wearing mouth–nose masks had become the main perceived limitation in daily life (Figure 1). Corresponding to the easing of measures, social activities (meeting family members and friends) and visits to doctors increased from Phase 1 to Phase 3 (Figure 2). Higher perceived limitations in daily living were associated with younger age (standardised beta = −1.9, $p = 0.001$) and earlier pandemic phase (standardised beta = −2.4, $p < 0.001$).

Furthermore, the perceived psychological burden decreased during the pandemic phases (Figure 3). Reasons, such as fear of infection, worries on how it should go on (insecurity and concerns) and reduced social contacts, for the psychological burden did not remarkably change during the phases (Figure 3). Higher psychological burden was associated with early pandemic phase (standardised beta = −2.6, p < 0.001), higher BDI (standardised beta = 0.18, $p = 0.001$) and higher MoCa (standardised beta = 0.11, $p = 0.06$).

From June 16, 2020, the people were also asked if they approve the easing of measures. Among the 136 interviewed patients, 67 (49.3%) approved, 55 (40.4) did not approve and 14 could not make a decision. The common reasons not to approve the easing of measures were fear of increased risk of infection and irresponsible behaviour of other people. Patients who did or did not approve easing of measures did not differ in terms of age ($p = 0.85$), gender ($p = 0.31$), education level ($p = 0.80$), marital state ($p = 0.52$), BDI ($p = 0.50$), TUG time ($p = 0.27$), MoCa ($p = 0.31$) and patient–physician relationship according to the HCCQ ($p = 0.44$). Both groups also did not differ in the perceived limitations in daily life ($p = 0.38$) and psychological burden ($p = 0.51$) caused by the measures.

For peer review only

DISCUSSION

Our study shows how perceived limitations of daily life declined with ongoing easing of measures and the reasons for this change during different pandemic phases. Perceived limitations due to the measures were higher at the beginning of the pandemic and in younger aged persons. There may be several reasons why older people perceived the limitations as less severe. For example, restrictions in the radius of action may be less relevant for people who were significantly restricted in their mobility than for younger and more active people. Furthermore, fewer responsibilities and more experiences with previous threatening situations can additionally explain the lower levels of perceived limitations among the elderly.[7] The most important limitation was the social contact restrictions. Even after various easing of the measures in Phase 3, these were still the main reasons for perceived limitations in everyday life. Although perceived limitations were associated with younger age, the elderly people are particularly susceptible to the effects of uncertainty and lack of social contacts.[8] After the easing of the measures, the most frequent reason for perceived limitations in everyday life was the use of the mouth–nose mask (46%). Overall, even though people reported discontent over the mandatory use of masks, the general population followed the rule relatively well, as wearing masks signals empathy and prosocial behaviour toward members of the risk group.[9] Parallel to the easing of the measures, social activities, such as visiting friends also increased.

On the psychological burden side, a relevant fear of coronavirus infection is indicated throughout all pandemic phases. This fear increased with the significant easing of measures in Phase 3. It is important to note here that the respondents were elderlies with various neurological and internal diseases. Many respondents stated that they were at increased risk of a severe course of COVID-19 disease and thus belonged to the risk group. This may explain the high level of anxiety.[5] Appropriately, approximately half of the respondents were critical on the easing of measures. However, the feeling of insecurity and worries about the future decreased over the course of the pandemic. This is the best indication that the respondents adapted to the specific requirements of the situation and were better informed than at the beginning of the pandemic, since sufficient information leads to less psychological burden like anxiety.[10]

For peer review only

The study has some limitations. It is important to emphasise that the data are cross-sectional, that is, different people were interviewed in the respective phases. Although the demographic and clinical parameters are comparable between the interviewed patients in the different phases, which is the usual design for other regular surveys in the context of the COVID-19 pandemic (e.g. COVID-19 Snapshot Monitoring (COSMO)[11]), distortions caused by this design cannot be excluded. Furthermore, the survey focused on elderly and sick people. Although this limits the generalizability of the results, these data are important because older people are often underrepresented in other surveys (e.g. COSMO).

For peer review only

FOOTNOTES

Acknowledgment: We thank Marieke Jäger and Verena Buchholz for assistance in data acquisition.

Author Contributions: AS, UT, HZ collected data, performed the statistical analysis and drafted the initial manuscript. TP provided the concept and design of the study and was involved in the analysis. HZ was involved in the interpretation of the data. TP revised the manuscript for important intellectual content. All authors read and approved the final manuscript.

Competing interests: None declared.

Ethics approval: All procedures performed in studies involving human participants were in accordance with the ethical standards of the institutional research committee (ethics committee of the Jena University Hospital, 4572-10/15) and with the 1964 Helsinki declaration and its later amendments or comparable ethical standards.

Funding: This work was supported by a Bundesministerium für Bildung und Forschung grant to Tino Prell (01GY1804). This funding source had no role in the design of this study and will not have any role during its execution, analyses, interpretation of the data, or decision to submit results.

Data sharing statement: Dataset is available as a supplement.

For peer review only

REFERENCES

1. Koo JR, Cook AR, Park M, et al. Interventions to mitigate early spread of SARS-CoV-2 in Singapore: a modelling study. *Lancet Infect Dis* 2020 doi: 10.1016/S1473-3099(20)30162-6
2. Prem K, Liu Y, Russell TW, et al. The effect of control strategies to reduce social mixing on outcomes of the COVID-19 epidemic in Wuhan, China: A modelling study. *Lancet Public Health* 2020 doi: 10.1016/S2468-2667(20)30073-6
3. Torales J, O'Higgins M, Castaldelli-Maia JM, et al. The outbreak of COVID-19 coronavirus and its impact on global mental health. *Int J Soc Psychiatry* 2020;66:317–20. doi: 10.1177/0020764020915212
4. Zipprich HM, Teschner U, Witte OW, et al. Knowledge, attitudes, practices, and burden during the COVID-19 pandemic in people with Parkinson's disease in Germany. *J Clin Med* 2020;9. doi: 10.3390/jcm9061643
5. Dubey S, Biswas P, Ghosh R, et al. Psychosocial impact of COVID-19. *Diabetes Metab Syndr* 2020;14:779–88. doi: 10.1016/j.dsx.2020.05.035
6. Prell T. Adherence to medication in neurogeriatric patients: an observational cross-sectional study. *BMC Public Health* 2019;19:1012. doi: 10.1186/s12889-019-7353-5
7. Ngo EB. When disasters and age collide: Reviewing vulnerability of the elderly. *Nat Hazards Rev* 2001;2:80–9. doi: doi:10.1061/(ASCE)1527-6988(2001)2:2(80)
8. Glowacz F, Schmits E. Psychological distress during the COVID-19 lockdown: The young adults most at risk. *Psychiatry Res* 2020;293:113486. doi: 10.1016/j.psychres.2020.113486 [published Online First: 2020/10/03]
9. Betsch C, Korn L, Sprengholz P, et al. Social and behavioral consequences of mask policies during the COVID-19 pandemic. *Proc Natl Acad Sci USA* 2020;117:21851–3. doi: 10.1073/pnas.2011674117
10. Rodríguez-Rey R, Garrido-Hernansaiz H, Collado S. Psychological impact and associated factors during the initial stage of the coronavirus (COVID-19) pandemic among the general population in Spain. *Front Psychol* 2020;11:1540. doi: 10.3389/fpsyg.2020.01540
11. Betsch C, Wieler L, Bosnjak M, et al. COVID-19 Snapshot Monitoring (COSMO): Monitoring knowledge, risk perceptions, preventive behaviours, and public trust in the current coronavirus outbreak. *Psych Archives* 2020 doi: 10.23668/psycharchives.2776

For peer review only

FIGURE LEGENDS

Figure 1. Change and reasons of perceived limitations in daily life during the COVID-19 pandemic.

Figure 2. Percentage of people performing different activities in different COVID-19 pandemic phases.

Figure 3. Change and reasons of psychological burden during the COVID-19 pandemic.

For peer review only

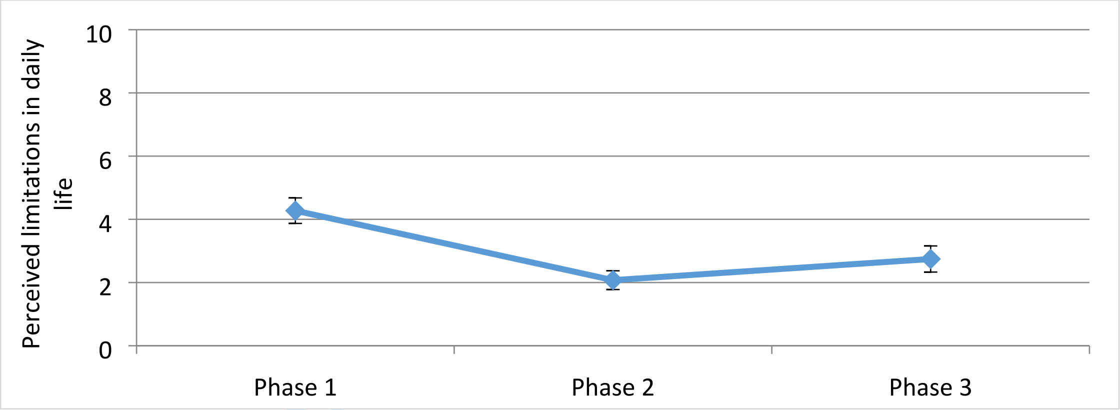

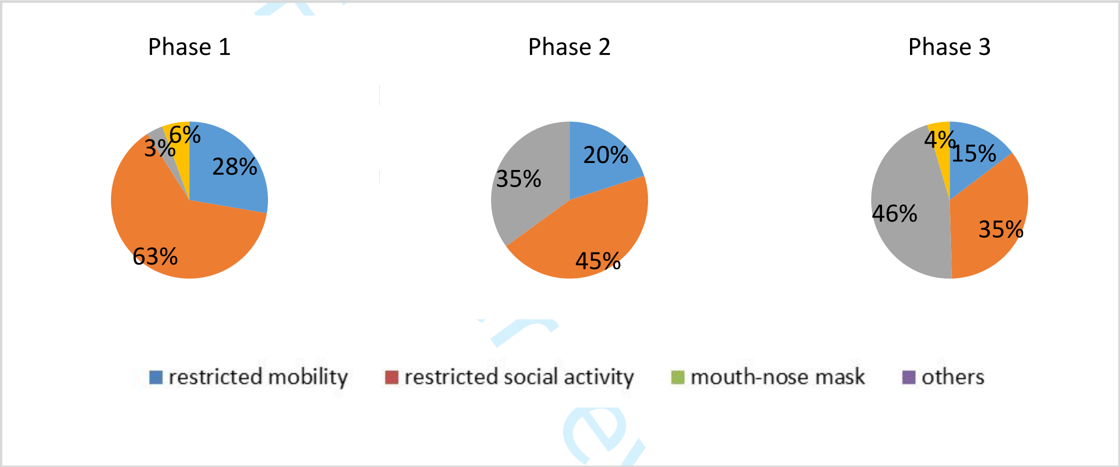

For peer review only

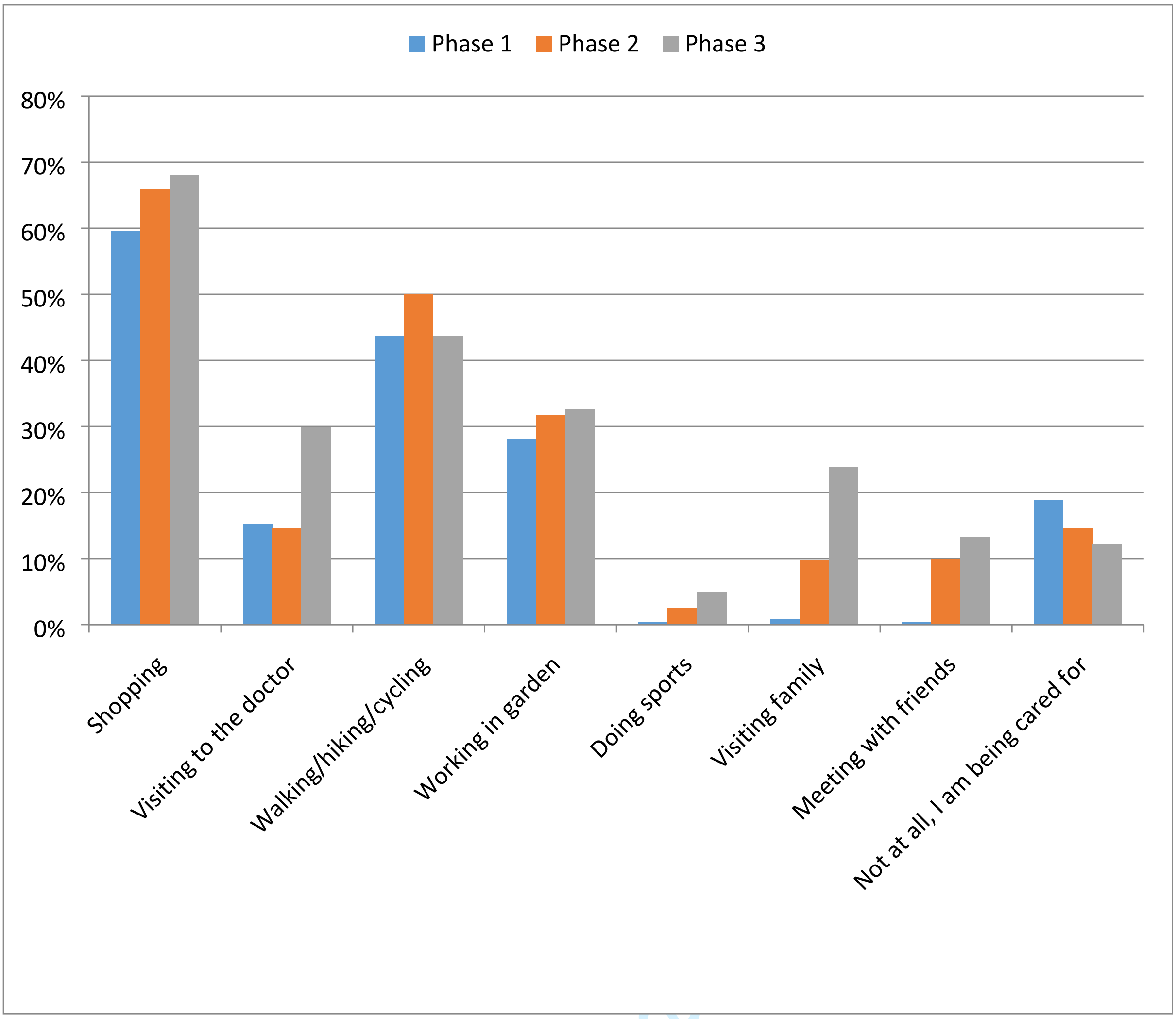
Phase 1
Phase 2
Phase 3
80%
70%
60%
50%
40%
30%
20%
10%
0%
Shopping
Visiting to the doctor
Walking/hiking/cycling
Working in garden
Doing sports
Visiting family
Meeting with friends
Not at all, I am being cared for

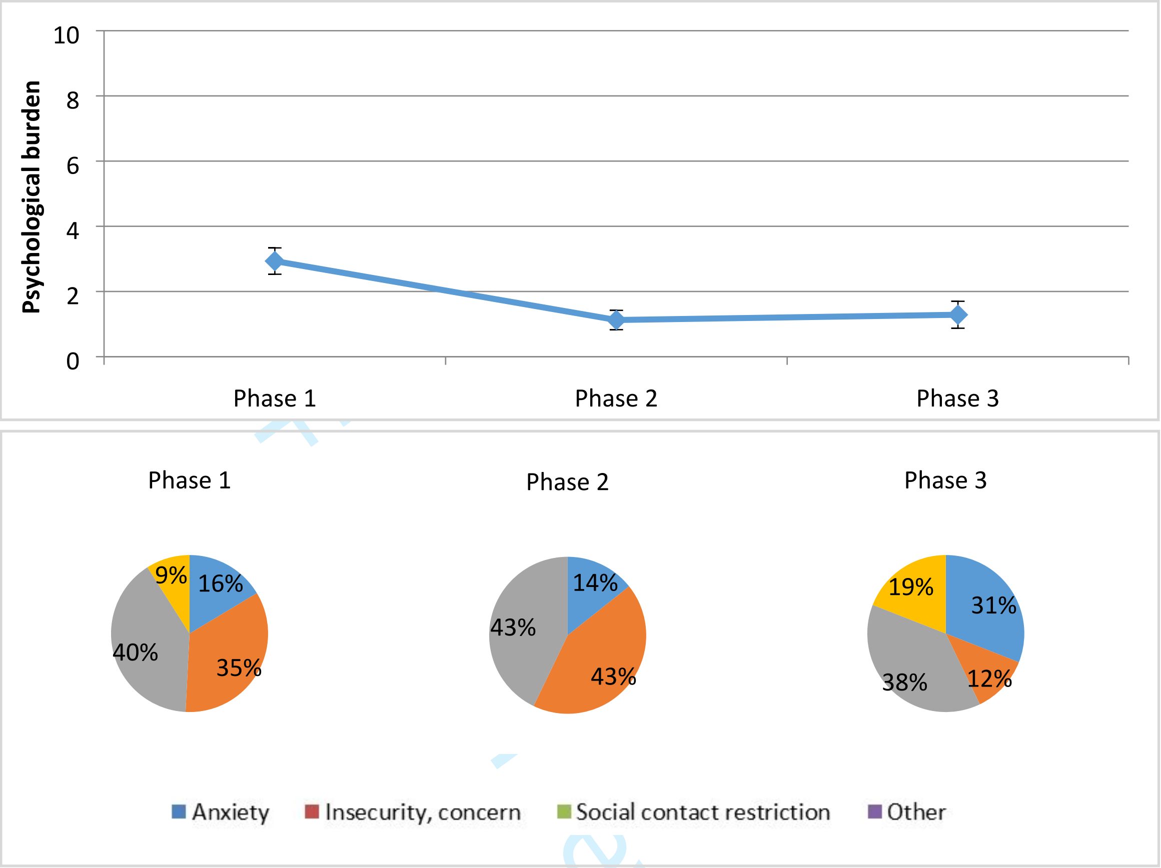
Psychological burden
10
8
6
4
2
0
Phase 1
Phase 2
Phase 3
Phase 1
Phase 2
Phase 3
9%
16%
40%
35%
14%
43%
43%
19%
31%
38%
12%
Anxiety
Insecurity, concern
Social contact restriction
Other

diag_group	Daily_life_	Daily_life_	Daily_life_	Daily_life_	Daily_life_	Daily_life_	Daily_life_	Daily_life_
movement	no	no	missing	no	no	no	no	yes
neuromusc	no	no	missing	no	no	no	no	yes
movement	no	no	yes	no	no	yes	yes	no
cerebrovas	yes	no	yes	no	yes	no	no	no
movement	yes	no	yes	no	yes	yes	yes	no
cerebrovas	yes	no	yes	yes	no	no	no	no
movement	no	no	yes	no	no	yes	no	no
movement	yes	no	yes	no	no	no	no	no
neuromusc	yes	no	yes	yes	no	yes	no	no
others	yes	no	yes	no	no	yes	yes	no
neuromusc	no	no	yes	yes	no	no	no	no
epilepsy	yes	no	yes	no	no	no	no	no
neuromusc	yes	no	yes	no	no	no	no	no
others	yes	no	yes	no	no	no	yes	no
cerebrovas	yes	yes	yes	no	no	no	no	no
others	yes	no	yes	no	no	no	no	no
neuromusc	yes	yes	yes	yes	no	yes	yes	no
others	yes	no	yes	no	no	no	no	no
cerebrovas	yes	no	yes	yes	no	no	no	no
cerebrovas	yes	no	yes	no	no	no	no	no
movement	no	no	yes	no	no	yes	yes	no
cerebrovas	yes	no	yes	yes	no	yes	yes	no
cerebrovas	yes	no	yes	yes	no	yes	no	no
cerebrovas	yes	yes	yes	no	no	yes	yes	no
cerebrovas	yes	yes	yes	yes	no	no	no	no
movement	yes	yes	yes	yes	yes	yes	yes	no
neuromusc	yes	yes	yes	no	no	yes	no	no
others	no	yes	yes	yes	no	no	no	no
others	yes	no	yes	yes	no	yes	no	no
others	yes	no	yes	yes	no	no	no	no
movement	yes	yes	yes	no	no	no	no	no
neuromusc	no	yes	yes	yes	no	missing	no	no
neuromusc	yes	no	yes	no	no	yes	yes	no
cerebrovas	yes	no	yes	no	no	no	no	no
cerebrovas	yes	no	yes	no	no	no	no	no
cerebrovas	yes	no	yes	yes	no	no	no	no
cerebrovas	yes	no	yes	yes	yes	no	yes	no
cerebrovas	yes	yes	yes	no	no	no	no	no
others	yes	no	yes	yes	no	no	no	no
others	no	no	yes	yes	no	no	no	no
cerebrovas	yes	yes	yes	no	no	no	no	no
cerebrovas	yes	no	yes	no	no	no	no	no
cerebrovas	yes	yes	yes	no	#NULL!	yes	yes	no
neuromusc	yes	no	yes	no	no	yes	yes	no
cerebrovas	yes	yes	yes	no	no	no	no	no
movement	yes	no	yes	yes	no	no	yes	no
neuromusc	no	no	yes	yes	no	no	yes	no
cerebrovas	yes	no	yes	yes	no	yes	yes	no
others	yes	no	yes	no	yes	yes	yes	no
movement	yes	no	yes	no	no	no	no	no
cerebrovas	yes	no	yes	yes	no	no	no	no

For peer review only

others	yes	no	yes	no	yes	yes	no	no
epilepsy	yes	no	yes	no	no	yes	no	no
movement	yes	no	yes	no	no	yes	yes	no
neuromusc	yes	yes	yes	no	no	no	no	no
movement	no	yes	yes	no	no	yes	no	no
movement	yes	no	yes	no	no	yes	no	no
others	yes	no	yes	no	no	yes	yes	no
movement	no	no	yes	no	yes	no	no	no
others	yes	no	yes	no	no	yes	no	no
movement	no	no	yes	yes	no	no	no	no
movement	yes	no	yes	no	no	no	no	no
others	yes	no	yes	no	no	no	no	no
neuromusc	yes	no	yes	no	no	yes	yes	no
others	yes	no	yes	no	no	no	no	no
cerebrovas	yes	no	yes	yes	no	no	no	no
neuromusc	no	no	yes	yes	no	no	no	no
movement	yes	no	yes	yes	no	no	no	no
cerebrovas	no	no	yes	yes	no	no	no	no
neuromusc	yes	yes	yes	yes	no	no	no	no
cerebrovas	yes	no	yes	no	no	no	no	no
movement	no	no	yes	no	yes	no	no	no
movement	yes	no	yes	yes	no	no	no	no
cerebrovas	no	no	yes	no	no	no	no	no
neuromusc	yes	no	yes	no	no	no	no	no
others	no	no	yes	no	no	no	no	no
others	no	no	yes	no	no	no	no	no
movement	no	no	yes	no	no	no	no	no
movement	yes	yes	yes	no	no	no	no	no
neuromusc	yes	no	yes	no	no	no	no	no
movement	yes	no	yes	no	no	no	no	no
others	yes	no	yes	no	no	no	no	no
movement	yes	no	yes	no	no	no	no	no
movement	yes	no	yes	no	no	no	no	no
cerebrovas	yes	no	yes	no	no	no	no	no
cerebrovas	no	no	yes	no	no	no	no	no
others	yes	no	yes	no	no	no	no	no
neuromusc	yes	no	yes	no	no	no	no	no
neuromusc	no	no	yes	no	no	no	no	no
others	yes	no	yes	no	no	no	no	no
movement	yes	no	yes	no	no	no	no	no
movement	no	no	yes	no	no	no	no	no
movement	yes	no	yes	yes	no	no	no	no
cerebrovas	yes	no	yes	no	no	no	no	no
neuromusc	yes	yes	yes	no	no	no	no	no
neuromusc	yes	yes	yes	yes	no	no	no	no
neuromusc	yes	no	yes	yes	no	no	no	no
movement	yes	no	yes	no	no	no	no	no
movement	no	no	yes	no	no	no	no	no
movement	yes	no	yes	no	no	no	no	no
cerebrovas	yes	no	yes	yes	no	no	no	no
neuromusc	yes	yes	yes	no	no	no	no	no
epilepsy	yes	no	yes	no	no	no	no	no

For peer review only

movement	yes	yes	yes	yes	no	no	no	no
others	no	no	yes	yes	no	no	no	no
cerebrovas	yes	yes	yes	no	no	no	no	no
neuromusc	yes	no	yes	yes	no	no	no	no
movement	yes	no	yes	no	no	no	no	no
movement	no	no	yes	no	no	no	no	no
cerebrovas	yes	no	yes	no	no	no	no	no
others	yes	yes	yes	yes	no	no	no	no
cerebrovas	yes	no	yes	no	no	no	no	no
movement	yes	no	yes	no	no	no	no	no
cerebrovas	yes	yes	yes	no	no	no	no	no
neuromusc	yes	no	yes	no	no	no	no	no
cerebrovas	yes	no	yes	no	no	no	no	no
movement	yes	yes	yes	no	no	no	no	no
others	yes	yes	yes	no	no	no	no	no
others	yes	yes	yes	no	no	no	no	no
neuromusc	yes	no	yes	yes	no	yes	no	no
neuromusc	no	no	yes	yes	no	no	no	no
neuromusc	yes	no	yes	no	no	no	no	no
others	yes	no	yes	no	no	no	no	no
neuromusc	yes	no	yes	no	no	no	no	no
others	yes	no	yes	no	no	no	no	no
others	no	no	yes	yes	no	no	no	no
cerebrovas	yes	no	yes	no	no	no	yes	no
others	yes	no	yes	yes	no	no	no	no
neuromusc	yes	no	yes	no	no	no	no	no
neuromusc	no	no	yes	yes	no	no	no	no
neuromusc	yes	no	yes	no	no	no	no	no
movement	yes	no	yes	no	no	no	no	no
movement	yes	yes	yes	no	no	no	no	no
neuromusc	no	no	yes	no	no	no	no	no
neuromusc	yes	no	yes	no	no	no	no	no
movement	yes	no	yes	no	no	yes	yes	no
movement	yes	no	yes	no	no	no	no	no
cerebrovas	yes	no	yes	no	no	no	no	no
cerebrovas	yes	no	yes	no	no	no	no	no
cerebrovas	yes	no	yes	no	no	no	missing	no
others	no	no	yes	no	no	no	no	no
movement	yes	no	yes	no	no	no	no	no
epilepsy	yes	no	yes	no	no	no	no	no
cerebrovas	no	no	yes	no	no	no	no	no
movement	no	yes	yes	no	no	no	no	no
movement	yes	no	yes	no	no	yes	no	no
movement	yes	no	yes	no	no	no	no	no
epilepsy	yes	no	yes	no	no	no	no	no
movement	yes	yes	yes	no	no	no	no	no
neuromusc	no	yes	yes	no	no	no	no	no
epilepsy	no	no	yes	no	no	no	no	no
cerebrovas	no	no	yes	no	no	no	no	no
neuromusc	yes	no	yes	no	no	missing	no	no
neuromusc	no	no	yes	no	no	no	no	no
neuromusc	no	no	yes	yes	no	no	no	no

For peer review only

neuromusc	yes	no	yes	no	no	no	no	no
cerebrovas	yes	no	yes	yes	no	no	no	no
others	yes	no	yes	no	no	no	no	no
neuromusc	yes	no	yes	no	no	no	no	no
movement	no	no	yes	no	no	no	no	no
movement	yes	yes	yes	no	no	no	no	no
neuromusc	yes	no	yes	no	no	no	no	no
movement	yes	no	yes	yes	no	no	no	no
neuromusc	yes	no	yes	no	no	no	no	no
cerebrovas	yes	no	yes	no	no	no	no	no
movement	yes	no	yes	yes	no	no	no	no
cerebrovas	yes	no	yes	no	no	no	no	no
cerebrovas	yes	no	yes	yes	no	no	no	no
movement	no	yes	yes	no	no	no	no	yes
cerebrovas	yes	yes	yes	no	no	no	no	no
cerebrovas	yes	yes	yes	no	no	no	no	no
others	no	yes	yes	yes	no	no	no	no
others	yes	yes	yes	no	no	no	no	no
movement	yes	no	yes	yes	no	no	no	no
neuromusc	no	no	yes	no	no	no	no	no
neuromusc	yes	no	yes	no	no	no	no	no
others	yes	no	yes	no	no	no	no	no
cerebrovas	no	no	yes	no	no	no	no	no
neuromusc	no	no	yes	yes	no	no	no	no
others	yes	no	yes	no	no	no	no	no
epilepsy	yes	no	yes	no	no	yes	yes	no
movement	yes	no	yes	no	no	no	no	no
epilepsy	yes	no	yes	yes	no	yes	no	no
cerebrovas	yes	no	yes	no	no	no	no	no
cerebrovas	no	no	yes	no	no	no	no	no
movement	yes	no	yes	no	no	yes	yes	no
movement	yes	no	yes	no	no	no	no	no
movement	yes	no	yes	yes	no	no	no	no
movement	yes	no	yes	yes	no	no	no	no
cerebrovas	no	no	yes	yes	no	no	no	no
others	no	no	yes	no	no	no	no	no
epilepsy	yes	no	yes	yes	no	no	no	no
cerebrovas	no	no	yes	no	no	no	no	no
cerebrovas	yes	no	yes	no	no	no	no	no
epilepsy	yes	no	yes	no	no	no	no	no
movement	yes	no	yes	yes	no	no	no	no
cerebrovas	yes	no	yes	no	no	no	no	no
movement	yes	no	yes	yes	no	no	no	no
cerebrovas	yes	no	yes	no	no	no	no	no
others	yes	no	yes	no	no	no	no	no
neuromusc	no	no	yes	yes	no	no	no	no
movement	no	no	no	no	no	no	no	no
cerebrovas	no	no	no	no	no	yes	no	yes
epilepsy	yes	yes	no	no	no	yes	no	no
epilepsy	no	no	no	yes	no	yes	no	no
others	no	yes	no	no	no	no	no	no
others	yes	no	no	no	no	no	no	no

For peer review only

movement	yes	no	no	no	no	no	no	no
cerebrovas	yes	no	no	yes	no	no	no	no
others	yes	no	no	no	no	no	no	no
movement	no	yes	no	no	no	no	no	no
movement	no	yes	no	no	no	no	no	no
neuromusc	no	yes	no	no	no	no	no	no
epilepsy	yes	no	no	no	no	no	no	no
cerebrovas	yes	no	no	no	no	no	no	no
others	no	no	no	no	no	no	no	yes
movement	yes	yes	no	no	no	no	no	no
movement	yes	yes	no	no	no	no	no	no
cerebrovas	yes	yes	no	no	no	yes	yes	no
cerebrovas	yes	no	no	yes	no	no	no	no
neuromusc	yes	no	no	yes	no	no	no	no
epilepsy	yes	yes	no	no	no	no	no	no
cerebrovas	yes	no	no	no	no	no	no	no
others	no	yes	no	no	no	no	no	no
others	no	yes	no	no	no	no	no	no
neuromusc	no	yes	no	yes	no	no	no	no
cerebrovas	no	yes	no	no	no	no	no	no
neuromusc	yes	yes	no	no	no	no	no	no
cerebrovas	no	no	no	no	no	no	no	no
neuromusc	no	no	no	no	no	no	no	yes
cerebrovas	yes	no	no	yes	no	no	no	no
movement	no	no	no	no	no	no	no	yes
cerebrovas	no	no	no	no	no	no	no	yes
cerebrovas	no	no	no	yes	no	no	no	no
neuromusc	yes	no	no	yes	no	no	no	no
cerebrovas	no	no	no	no	yes	no	no	no
epilepsy	yes	no	no	no	no	no	no	no
neuromusc	yes	no	no	no	no	no	no	no
cerebrovas	yes	yes	no	no	no	yes	no	no
neuromusc	no	no	no	no	no	no	no	yes
movement	yes	no	no	no	no	no	no	no
movement	no	yes	no	no	no	no	no	no
neuromusc	yes	no	no	no	no	no	no	no
movement	yes	no	no	no	no	no	no	no
movement	yes	no	no	no	no	no	no	no
movement	yes	no	no	yes	no	no	no	no
epilepsy	yes	yes	no	yes	no	no	no	no
cerebrovas	no	no	no	no	no	no	no	yes
neuromusc	no	no	no	yes	no	no	no	no
cerebrovas	yes	yes	no	no	no	yes	no	no
cerebrovas	yes	no	no	yes	no	no	no	no
neuromusc	yes	no	no	no	no	no	no	no
cerebrovas	yes	no	no	no	no	yes	no	no
cerebrovas	no	yes	no	yes	no	no	no	yes
others	no	no	no	no	no	no	no	yes
neuromusc	yes	yes	no	yes	no	no	no	no
neuromusc	yes	yes	no	yes	no	no	no	no
cerebrovas	no	yes	no	no	no	no	no	yes
cerebrovas	no	yes	no	yes	no	no	no	no

For peer review only

neuromusc	no	yes	no	no	no	yes	no	no
cerebrovas	no	yes	no	no	no	no	no	no
cerebrovas	yes	no	no	yes	no	yes	no	no
others	no	no	no	no	no	no	no	yes
cerebrovas	yes	no	no	no	no	no	no	no
cerebrovas	yes	no	no	no	no	no	no	no
cerebrovas	yes	no	no	yes	no	no	no	no
epilepsy	yes	no	no	no	no	no	no	no
others	no	no	no	no	no	no	no	yes
movement	yes	no	no	no	yes	yes	yes	no
others	yes	no	no	no	no	no	no	no
movement	yes	no	no	yes	no	no	no	no
cerebrovas	yes	no	no	yes	no	yes	no	no
movement	yes	no	no	no	no	yes	yes	no
cerebrovas	no	yes	no	no	no	no	no	yes
cerebrovas	no	no	no	no	no	no	no	yes
others	no	no	no	yes	no	no	no	no
neuromusc	yes	no	no	no	no	no	no	no
movement	yes	no	no	yes	no	no	no	no
movement	yes	no	no	yes	no	no	no	no
neuromusc	yes	no	no	no	no	no	no	no
movement	yes	yes	no	no	no	no	no	no
others	yes	no	no	yes	no	no	no	no
movement	yes	no	no	no	no	yes	no	no
movement	yes	no	no	yes	no	no	no	no
others	yes	no	no	yes	yes	no	yes	no
neuromusc	no	no	no	no	no	no	no	yes
neuromusc	yes	no	no	no	no	no	no	no
cerebrovas	yes	no	no	yes	no	no	no	no
neuromusc	yes	no	no	no	no	no	no	no
neuromusc	yes	no	no	yes	no	no	no	no
movement	yes	no	no	no	no	no	no	no
others	yes	no	no	yes	no	no	no	no
neuromusc	yes	no	no	no	no	no	no	no
movement	no	no	no	no	no	missing	missing	yes
movement	yes	no	no	no	no	no	no	no
others	yes	yes	no	no	no	no	no	no
cerebrovas	yes	no	no	yes	no	no	no	no
neuromusc	yes	no	no	no	no	no	no	no
movement	yes	yes	no	no	no	no	no	no
neuromusc	no	yes	no	yes	no	yes	no	no
movement	no	no	no	no	no	no	no	yes
neuromusc	yes	no	no	no	no	no	no	no
cerebrovas	no	no	no	missing	no	no	no	yes
movement	yes	no	no	yes	no	no	no	no
movement	no	no	no	no	no	no	no	yes
neuromusc	yes	no	no	yes	no	no	no	no
neuromusc	yes	yes	no	no	no	no	no	no
others	yes	no	no	no	no	no	no	no
cerebrovas	no	no	no	no	no	no	no	yes
others	yes	no	no	no	no	no	no	no
others	no	no	no	no	no	no	no	yes

For peer review only

others	no	no	no	no	no	no	no	no
cerebrovas	yes	no	no	yes	no	no	no	no
movement	no	yes	no	no	no	no	no	no
cerebrovas	no	no	no	yes	no	no	no	no
neuromusc	yes	no	no	yes	no	no	no	no
neuromusc	no	no	no	no	no	no	no	yes
neuromusc	yes	no	no	no	no	no	no	no
epilepsy	yes	no	no	yes	no	no	no	no
neuromusc	yes	no	no	yes	no	no	no	no
neuromusc	no	no	no	no	no	no	no	yes
neuromusc	no	no	no	no	no	no	no	yes
movement	no	no	no	no	no	no	no	yes
neuromusc	yes	yes	no	no	no	no	no	no
cerebrovas	yes	no	no	yes	no	no	no	no
neuromusc	no	yes	no	no	no	no	no	no
others	no	no	no	yes	no	no	no	yes
others	yes	yes	no	no	no	no	no	no
neuromusc	no	no	no	no	no	no	no	no
epilepsy	yes	no	no	no	no	no	no	no
movement	no	no	no	no	no	no	no	yes
movement	no	no	no	yes	no	no	no	yes
neuromusc	no	no	no	no	no	no	no	yes
movement	no	yes	no	yes	no	no	no	no
cerebrovas	no	no	no	no	no	no	no	yes
cerebrovas	yes	no	no	yes	no	no	no	no
others	no	no	no	no	no	no	no	yes
movement	no	no	no	yes	no	no	no	no
cerebrovas	yes	yes	no	no	no	no	no	no
neuromusc	yes	yes	no	yes	no	no	no	no
cerebrovas	no	no	no	no	no	no	no	yes
movement	yes	no	no	no	no	no	no	#NULL!
movement	yes	no	no	yes	no	no	no	no
cerebrovas	yes	no	no	no	no	no	no	no
neuromusc	yes	yes	no	no	no	no	no	no
neuromusc	no	no	no	no	no	no	no	yes
movement	yes	no	no	no	no	no	no	no
others	yes	yes	no	no	no	no	no	no
cerebrovas	no	no	no	yes	no	no	no	yes
movement	yes	no	no	no	no	no	no	no
others	yes	no	no	no	no	no	yes	no
neuromusc	yes	yes	no	no	no	no	no	no
neuromusc	yes	no	no	yes	no	no	no	no
movement	yes	no	no	yes	no	no	no	no
epilepsy	yes	no	no	yes	no	no	no	no
movement	yes	no	no	yes	no	no	no	no
others	yes	no	no	no	no	no	no	no
cerebrovas	no	no	no	yes	no	no	no	no
movement	no	no	no	no	no	no	no	yes
movement	yes	no	no	yes	no	no	no	no
neuromusc	no	no	no	no	no	no	no	yes
neuromusc	no	no	no	no	no	no	no	yes
neuromusc	yes	yes	no	no	no	yes	no	no

For peer review only

others	no	yes	no	yes	no	no	no	no
neuromusc	yes	yes	no	no	no	no	no	no
epilepsy	yes	no	no	no	no	yes	no	no
cerebrovas	no	no	no	no	no	no	no	yes
movement	no	no	no	yes	no	no	no	no
movement	yes	no	no	no	no	yes	no	no
others	yes	no	no	no	no	no	no	no
others	no	yes	no	yes	no	no	no	no
movement	no	no	no	no	no	no	no	yes
cerebrovas	yes	no	no	no	no	no	no	no
cerebrovas	no	no	no	yes	no	no	no	no
movement	no	no	no	no	no	no	no	yes
others	no	no	no	yes	no	no	no	no
neuromusc	yes	no	no	missing	no	no	no	no
neuromusc	no	no	no	yes	no	no	no	no
movement	no	no	no	no	no	no	no	yes
others	yes	yes	no	no	no	no	no	no
others	yes	no	no	no	no	no	no	no
others	no	yes	no	yes	no	no	no	no
others	yes	no	no	yes	no	no	no	no
cerebrovas	yes	yes	no	no	no	no	no	no
cerebrovas	yes	no	no	no	no	no	no	no
neuromusc	yes	no	no	no	no	no	no	no
movement	no	no	no	no	no	no	no	yes
movement	no	no	no	no	no	no	no	yes
neuromusc	yes	no	no	yes	no	no	no	no
neuromusc	yes	no	no	yes	no	no	no	no
cerebrovas	no	no	no	no	no	no	no	yes
movement	no	missing	no	no	no	missing	no	yes
others	yes	yes	no	no	no	no	no	no
movement	no	yes	no	no	no	no	no	no
cerebrovas	no	yes	no	no	no	no	no	no
movement	no	yes	no	no	no	no	no	no
movement	yes	yes	no	yes	no	no	no	no
others	no	yes	no	no	no	no	no	no
neuromusc	no	yes	no	no	no	no	no	yes
cerebrovas	no	yes	no	no	no	no	no	no
cerebrovas	yes	yes	no	yes	no	no	no	no
movement	yes	no	no	no	no	no	no	no
others	yes	no	no	yes	no	no	no	no
cerebrovas	no	no	no	no	no	no	no	no
neuromusc	no	no	no	no	no	no	no	yes
others	yes	no	no	no	no	no	no	no
others	no	no	no	yes	no	no	no	no
others	no	no	no	no	no	no	no	yes
neuromusc	yes	no	no	no	no	no	no	no
others	yes	no	no	no	no	no	no	no
movement	no	no	no	no	no	no	no	yes
cerebrovas	yes	no	no	no	no	no	no	no
cerebrovas	yes	no	no	no	no	no	no	no
cerebrovas	no	no	no	no	no	no	no	yes
neuromusc	no	no	no	no	no	no	no	yes

For peer review only

cerebrovas	no	no	no	no	no	no	no	yes
others	no	no	no	yes	no	no	no	no
neuromusc	yes	no	no	no	no	no	no	no
neuromusc	yes	no	no	no	no	no	no	no
neuromusc	no	no	no	no	no	no	no	yes
movement	no	no	no	no	no	no	no	yes
cerebrovas	yes	no	no	yes	no	no	no	no
cerebrovas	yes	no	no	no	no	no	no	no
neuromusc	no	no	no	no	no	no	no	yes
epilepsy	no	no	no	no	no	no	no	yes
movement	no	no	no	no	no	no	no	no
movement	no	no	no	no	no	no	no	no
movement	yes	no	no	yes	no	no	no	no
movement	no	no	no	no	no	no	no	yes
movement	yes	no	no	yes	no	no	no	no
cerebrovas	no	no	no	no	no	no	no	yes
cerebrovas	yes	no	no	no	no	no	no	no
others	yes	no	no	yes	no	no	no	no
cerebrovas	no	no	no	yes	no	no	no	yes
others	yes	no	no	yes	no	no	no	no
epilepsy	yes	no	no	no	no	no	no	no
neuromusc	no	no	no	no	no	no	no	yes
movement	no	no	no	no	no	no	no	yes
neuromusc	no	no	no	no	no	no	no	yes
movement	no	no	no	yes	no	no	no	no
cerebrovas	no	no	no	no	no	no	no	yes
movement	no	no	no	no	no	no	no	yes
movement	no	no	no	yes	no	no	no	yes
movement	yes	no	no	no	no	no	no	no
epilepsy	no	no	no	yes	no	no	no	yes
movement	no	no	no	no	no	no	no	yes
movement	no	no	no	yes	no	no	no	no
cerebrovas	yes	no	no	no	no	no	no	no
movement	no	no	no	yes	no	no	no	no
others	yes	yes	no	no	no	no	no	no
others	no	no	no	no	no	no	no	yes
others	yes	no	no	no	no	no	no	no

For peer review only

Daily_life_l	Psychologi	Phase	Approval_e	BDI_II	HCCQ	MoCa	TuG_sec
5.0	5.0	1	not asked	3	6.6	21	7
0	0	2	not asked	7	missing	27	not performed due to i
1.0	0	3	3	1	5.7	25	10
1.5	0	3	3	0	6.9	28	not performed due to i
2.0	0	3	3	7	6.7	30	6
2.5	0	3	3	9	6.6	25	10
3.0	0	3	3	25	3.8	16	not performed due to i
4.0	0	3	3	16	6.3	22	15
5.0	0	3	3	4	6.5	18	not performed due to i
6.0	0	3	3	5	7.0	19	not performed due to i
0	0	3	3	3	5.9	18	26
0	0	3	3	3	6.6	20	not performed due to i
0	0	3	3	27	2.4	25	not performed due to i
4.0	3.0	3	no	16	missing	24	not performed due to i
5.0	3.0	3	no	8	7.0	20	not performed due to i
1.0	4.0	3	no	13	missing	25	not performed due to i
7.0	4.0	3	no	5	6.1	27	6
4.0	5.0	3	no	3	6.1	30	7
2.0	5.5	3	no	10	1.0	16	not performed due to i
3.0	8.0	3	no	3	6.7	21	not performed due to i
1.0	0	3	no	9	6.4	21	10
2.0	0	3	no	13	1.5	19	not performed due to i
2.0	0	3	no	9	7.0	28	9
2.5	0	3	no	15	5.5	27	not performed due to i
3.0	0	3	no	11	5.7	25	not performed due to i
3.0	0	3	no	4	5.9	23	8
4.0	0	3	no	11	6.1	24	8
5.0	0	3	no	23	7.0	21	not performed due to i
5.0	0	3	no	11	6.0	26	7
7.0	0	3	no	7	3.1	missing	not performed due to i
0	0	3	no	4	6.0	20	7
0	0	3	no	9	5.1	19	13
0	0	3	no	4	5.8	20	12
0	0	3	no	2	6.4	29	7
0	0	3	no	3	6.5	22	8
0	0	3	no	0	6.5	18	9
0	0	3	no	4	6.6	26	11
5.0	5.0	3	yes	11	5.1	23	not performed due to i
5.0	5.0	3	yes	11	6.2	24	8
5.0	5.0	3	yes	24	3.0	11	9
1.0	0	3	yes	1	7.0	missing	not performed due to i
1.0	0	3	yes	13	3.8	missing	9
1.5	0	3	yes	15	6.7	29	not performed due to i
1.5	0	3	yes	8	6.3	24	7
2.0	0	3	yes	6	6.6	23	9
2.0	0	3	yes	30	missing	28	8
2.0	0	3	yes	8	6.5	27	10
2.0	0	3	yes	4	6.7	25	not performed due to i
3.0	0	3	yes	13	6.7	24	8
3.0	0	3	yes	4	4.7	24	13
3.5	0	3	yes	1	missing	20	12

For peer review only

4.0	0	3	yes	6	5.9	27	7
5.0	0	3	yes	3	5.9	25	6
8.0	0	3	yes	1	7.0	23	10
0	0	3	yes	4	6.8	24	9
0	0	3	yes	6	7.0	18	not performed due to i
0	0	3	yes	5	6.7	20	12
0	0	3	yes	5	7.0	26	6
0	0	3	yes	18	5.1	20	7
0	0	3	yes	14	7.0	25	8
0	0	3	yes	2	6.6	25	9
0	0	3	yes	11	5.3	21	11
0	10	3	yes	15	5.7	28	8
10	10	3	yes	14	4.5	28	9
10	1.0	1	not asked	14	6.3	27	9
1.0	2.0	1	not asked	1	missing	24	7
2.0	2.0	1	not asked	2	missing	23	7
2.0	2.0	1	not asked	33	2.5	21	9
5.0	2.0	1	not asked	10	4.7	23	10
3.0	3.0	1	not asked	0	6.9	missing	7
4.0	3.0	1	not asked	8	missing	28	9
10	3.0	1	not asked	5	6.5	18	7
3.0	3.5	1	not asked	8	6.1	23	10
5.0	3.5	1	not asked	6	7.0	13	not performed due to i
4.0	4.0	1	not asked	12	7.0	28	11
5.0	4.0	1	not asked	6	6.6	19	16
5.0	4.0	1	not asked	11	missing	0	17
5.0	4.0	1	not asked	12	6.8	20	15
6.5	4.5	1	not asked	0	6.1	26	6
2.0	5.0	1	not asked	20	4.8	25	7
3.5	5.0	1	not asked	9	7.0	29	7
4.0	5.0	1	not asked	5	5.9	24	9
4.0	5.0	2	not asked	33	1.4	22	not performed due to i
4.5	5.0	1	not asked	13	6.7	27	not performed due to i
5.0	5.0	1	not asked	2	7.0	19	8
5.0	5.0	1	not asked	7	6.1	22	11
5.5	5.0	1	not asked	10	6.3	27	not performed due to i
6.0	5.0	1	not asked	3	4.7	25	7
7.0	5.0	1	not asked	15	5.9	27	not performed due to i
7.0	5.0	1	not asked	11	5.5	22	8
7.0	5.0	1	not asked	11	6.7	22	9
7.5	5.0	1	not asked	10	missing	22	9
8.0	5.0	1	not asked	13	7.0	19	10
8.0	5.0	1	not asked	3	4.8	25	7
10	5.0	1	not asked	11	6.9	17	19
10	5.0	1	not asked	1	6.7	24	11
8.5	5.5	1	not asked	7	missing	26	11
6.0	6.0	1	not asked	37	5.8	17	35
5.0	6.5	1	not asked	9	7.0	28	6
5.0	6.5	1	not asked	21	6.0	16	not performed due to i
5.5	6.5	1	not asked	15	6.7	20	not performed due to i
6.5	6.5	1	not asked	19	5.7	23	11
8.0	6.5	1	not asked	10	4.7	21	7

For peer review only

8.5	6.5	1	not asked	29	6.8	25	9
4.0	7.0	1	not asked	12	4.9	26	11
4.5	7.0	3	not asked	6	6.9	16	not performed due to i
5.0	7.0	1	not asked	6	6.0	22	20
5.0	7.0	1	not asked	18	6.4	23	8
5.0	7.0	1	not asked	8	missing	27	8
6.0	7.0	1	not asked	11	6.2	28	7
7.0	7.0	1	not asked	38	3.3	24	11
7.0	7.0	1	not asked	16	7.0	22	7
8.0	7.0	1	not asked	17	2.9	24	11
7.5	7.5	1	not asked	16	2.3	20	10
4.0	8.0	1	not asked	4	6.2	24	7
5.0	8.0	1	not asked	7	6.8	29	9
6.0	8.0	1	not asked	31	6.2	16	5
8.0	8.0	1	not asked	17	7.0	28	7
8.0	8.0	2	not asked	16	6.8	27	not performed due to i
8.0	8.0	3	not asked	3	6.0	20	not performed due to i
0	8.0	1	not asked	missing	missing	missing	not performed due to i
5.5	8.5	2	not asked	6	7.0	26	6
0	8.5	1	not asked	3	6.5	26	7
5.0	9.0	1	not asked	15	2.5	29	18
1.0	0	1	not asked	25	6.3	29	not performed due to i
1.0	0	2	not asked	4	3.2	30	19
2.0	0	2	not asked	7	3.6	21	not performed due to i
2.0	0	3	not asked	9	3.5	25	8
2.0	0	3	not asked	1	7.0	29	7
2.0	0	1	not asked	6	7.0	26	7
2.0	0	1	not asked	9	5.6	24	7
2.5	0	1	not asked	7	7.0	25	10
3.0	0	1	not asked	16	5.1	24	8
3.0	0	1	not asked	29	6.0	25	23
3.0	0	1	not asked	5	6.7	26	7
3.0	0	3	not asked	13	6.9	25	9
3.0	0	1	not asked	7	5.8	20	8
3.0	0	1	not asked	4	7.0	19	not performed due to i
3.0	0	1	not asked	4	6.5	27	not performed due to i
3.0	0	2	not asked	6	5.1	26	not performed due to i
3.0	0	2	not asked	13	2.7	22	12
3.5	0	1	not asked	9	7.0	29	7
3.5	0	3	not asked	8	5.2	23	not performed due to i
3.5	0	1	not asked	7	7.0	27	10
4.0	0	1	not asked	12	5.1	29	not performed due to i
4.0	0	2	not asked	7	4.6	21	9
4.0	0	3	not asked	9	3.5	18	15
4.5	0	1	not asked	5	missing	23	7
5.0	0	2	not asked	7	5.9	23	7
5.0	0	3	not asked	4	7.0	17	10
5.0	0	1	not asked	1	6.3	22	9
5.0	0	1	not asked	9	6.8	missing	12
5.0	0	1	not asked	2	6.6	23	11
5.0	0	1	not asked	3	6.9	missing	14
5.0	0	1	not asked	14	6.0	27	10

For peer review only

5.0	0	1	not asked	0	4.5	25	7
5.0	0	1	not asked	3	5.7	27	not performed due to i
5.0	0	1	not asked	5	missing	21	10
5.0	0	3	not asked	4	6.7	26	not performed due to i
5.0	0	1	not asked	15	4.3	23	9
6.5	0	3	not asked	4	4.6	25	7
6.5	0	2	not asked	13	6.1	28	7
7.0	0	1	not asked	6	6.9	missing	8
8.0	0	1	not asked	8	5.6	26	8
8.0	0	1	not asked	3	7.0	28	9
8.0	0	1	not asked	11	6.3	24	7
8.0	0	1	not asked	0	5.0	25	not performed due to i
8.0	0	1	not asked	3	4.3	17	not performed due to i
0	0	1	not asked	0	7.0	21	10
0	0	3	not asked	1	7.0	21	not performed due to i
0	0	3	not asked	14	6.6	24	10
0	0	3	not asked	10	7.0	22	not performed due to i
0	0	3	not asked	8	6.6	25	10
0	0	1	not asked	5	6.6	18	14
0	0	1	not asked	24	4.7	25	9
0	0	1	not asked	8	3.5	28	10
0	0	1	not asked	17	missing	21	10
0	0	1	not asked	13	6.3	26	9
0	0	1	not asked	7	6.9	26	7
0	0	2	not asked	8	6.2	15	not performed due to i
0	0	2	not asked	1	3.1	6	not performed due to i
0	0	2	not asked	3	6.1	29	10
0	0	3	not asked	0	6.9	1	14
0	0	3	not asked	4	7.0	27	17
0	0	1	not asked	1	missing	24	not performed due to i
0	0	1	not asked	15	6.9	23	11
0	0	1	not asked	1	4.3	25	9
0	0	2	not asked	13	6.6	missing	not performed due to i
0	0	2	not asked	7	6.2	23	7
0	0	3	not asked	0	7.0	missing	not performed due to i
0	0	1	not asked	11	5.8	10	8
0	0	1	not asked	0	6.0	27	not performed due to i
0	0	1	not asked	19	3.6	21	not performed due to i
0	0	1	not asked	7	5.5	15	not performed due to i
0	0	1	not asked	18	6.9	missing	7
0	0	2	not asked	2	6.6	25	not performed due to i
0	0	2	not asked	44	6.4	20	9
0	0	2	not asked	3	6.7	27	7
0	0	2	not asked	3	7.0	26	not performed due to i
0	0	2	not asked	6	5.9	25	11
7.5	10	1	not asked	8	6.5	25	10
3.0	3.0	3	3	12	missing	21	10
0	0	3	3	6	missing	21	12
10	0	3	3	10	5.7	18	8
6.5	3.5	3	no	6	5.8	27	7
3.0	4.0	3	no	8	6.3	24	7
5.0	5.0	3	no	7	missing	24	not performed due to i

For peer review only

5.0	5.0	3	no	25	6.3	20	11
8.0	5.0	3	no	9	6.1	24	not performed due to i
8.0	8.0	3	no	7	5.5	29	9
0	8.0	3	no	19	4.0	30	11
10	8.0	3	no	6	6.5	21	10
8.5	8.5	3	no	19	7.0	24	9
1.5	0	3	no	12	7.0	24	10
2.0	0	3	no	13	4.4	25	not performed due to i
2.0	0	3	no	22	5.7	27	17
4.0	0	3	no	12	4.9	17	12
5.0	0	3	no	5	5.1	20	10
5.0	0	3	no	5	6.4	20	8
5.0	0	3	no	12	5.7	23	10
5.0	0	3	no	9	4.0	26	8
6.0	0	3	no	7	6.6	25	not performed due to i
7.0	0	3	no	14	5.5	26	10
8.0	0	3	no	5	6.7	missing	not performed due to i
0	0	3	no	16	5.4	24	11
0	0	3	no	4	5.9	24	9
0	0	3	no	11	6.9	25	not performed due to i
0	0	3	no	4	7.0	20	9
0	0	3	no	7	6.9	22	11
0	0	3	no	1	6.9	26	not performed due to i
0	0	3	no	5	6.9	26	not performed due to i
0	0	3	no	11	6.9	24	22
0	0	3	no	35	4.9	28	11
0	0	3	no	15	4.0	17	22
0	0	3	no	3	6.2	missing	7
3.0	2.0	3	yes	14	4.1	25	8
3.0	3.0	3	yes	5	6.5	22	10
4.0	3.0	3	yes	8	5.9	19	8
8.0	3.0	3	yes	9	7.0	16	11
5.0	5.0	3	yes	5	6.1	22	not performed due to i
5.0	5.0	3	yes	11	3.4	24	5
7.0	5.0	3	yes	21	missing	28	10
8.5	5.0	3	yes	0	6.8	27	not performed due to i
6.0	6.0	3	yes	12	2.7	22	10
4.0	7.0	3	yes	15	5.5	27	10
1.0	0	3	yes	9	6.9	28	5
2.0	0	3	yes	15	7.0	21	9
2.0	0	3	yes	5	6.0	17	not performed due to i
2.5	0	3	yes	6	missing	24	10
3.0	0	3	yes	13	7.0	22	not performed due to i
3.0	0	3	yes	2	7.0	23	not performed due to i
3.0	0	3	yes	5	6.2	26	not performed due to i
4.0	0	3	yes	3	missing	26	9
5.0	0	3	yes	4	7.0	20	not performed due to i
6.5	0	3	yes	13	6.2	18	not performed due to i
0	0	3	yes	0	6.5	22	6
0	0	3	yes	8	6.5	25	not performed due to i
0	0	3	yes	8	6.1	missing	not performed due to i
0	0	3	yes	3	6.0	23	not performed due to i

For peer review only

0	0	3	yes	10	6.5	28	not performed due to i
0	0	3	yes	8	missing	22	24
0	0	3	yes	29	5.0	26	7
0	0	3	yes	3	5.7	16	not performed due to i
0	0	3	yes	7	6.5	missing	not performed due to i
0	0	3	yes	11	6.6	20	16
0	0	3	yes	0	6.6	13	not performed due to i
0	0	3	yes	9	6.9	25	9
0	0	3	yes	11	5.1	23	9
0	0	3	yes	7	5.7	14	12
0	0	3	yes	8	5.9	25	7
0	0	3	yes	6	missing	missing	8
0	0	3	yes	10	6.1	27	not performed due to i
0	0	3	yes	4	3.8	24	9
10	0	3	yes	14	5.7	12	not performed due to i
10	10	3	yes	10	6.1	18	not performed due to i
2.0	1.0	1	not asked	6	5.9	26	not performed due to i
3.0	1.5	1	not asked	2	6.7	24	9
2.0	2.0	3	not asked	18	5.3	28	8
4.0	2.0	1	not asked	1	5.6	27	15
5.0	2.0	1	not asked	11	5.8	27	25
6.0	2.0	1	not asked	18	4.9	22	not performed due to i
2.0	2.5	1	not asked	8	7.0	28	8
10	2.5	1	not asked	6	6.7	18	12
2.0	3.0	1	not asked	19	6.1	27	7
3.0	3.0	2	not asked	2	6.7	29	6
5.0	3.0	1	not asked	2	6.5	20	not performed due to i
7.0	3.0	1	not asked	2	5.5	24	9
8.0	3.0	1	not asked	6	6.1	23	11
3.5	3.5	1	not asked	5	5.8	26	12
5.0	3.5	1	not asked	3	6.8	missing	13
5.0	3.5	1	not asked	12	6.7	25	8
6.5	3.5	1	not asked	8	6.5	23	12
2.0	4.0	1	not asked	11	5.2	25	8
4.0	4.0	1	not asked	10	3.9	21	not performed due to i
5.0	4.0	1	not asked	2	7.0	missing	9
0	4.0	1	not asked	9	missing	26	8
5.0	4.5	1	not asked	4	6.0	27	13
2.0	5.0	3	not asked	29	5.4	25	12
5.0	5.0	3	not asked	23	4.3	17	20
5.0	5.0	3	not asked	7	6.9	28	10
5.0	5.0	1	not asked	13	5.5	22	not performed due to i
5.0	5.0	1	not asked	6	6.5	24	37
5.0	5.0	1	not asked	4	4.7	20	not performed due to i
6.0	5.0	1	not asked	10	6.5	30	8
6.0	5.0	1	not asked	7	5.6	26	20
6.0	5.0	1	not asked	11	6.4	24	8
7.5	5.0	1	not asked	6	5.3	19	14
8.0	5.0	1	not asked	0	7.0	27	10
8.0	5.0	1	not asked	8	5.8	missing	not performed due to i
8.0	5.0	1	not asked	0	6.6	31	13
8.0	5.0	1	not asked	14	6.0	29	30

For peer review only

0	5.0	1	not asked	3	6.0	missing	not performed due to i
10	5.0	1	not asked	2	6.3	26	7
1.0	5.5	1	not asked	21	5.7	9	not performed due to i
5.5	5.5	1	not asked	18	4.9	25	not performed due to i
6.5	5.5	1	not asked	8	5.1	26	11
8.0	5.5	3	not asked	8	5.7	25	not performed due to i
10	5.5	1	not asked	16	6.3	27	15
5.0	6.0	2	not asked	2	6.3	18	12
8.0	6.0	1	not asked	12	5.2	29	7
6.5	6.5	3	not asked	8	3.7	22	not performed due to i
9.0	6.5	2	not asked	12	6.7	24	not performed due to i
5.0	7.0	1	not asked	10	5.9	24	not performed due to i
6.0	7.0	1	not asked	2	5.9	26	11
6.0	7.0	1	not asked	0	6.5	missing	10
7.0	7.0	1	not asked	20	4.7	25	12
7.0	7.0	1	not asked	12	6.8	19	10
7.5	7.0	1	not asked	16	6.1	26	not performed due to i
8.0	7.0	1	not asked	12	5.4	25	7
8.0	7.0	1	not asked	34	5.3	25	10
8.0	7.5	1	not asked	14	2.4	21	27
8.5	7.5	1	not asked	24	5.8	19	not performed due to i
1.0	8.0	1	not asked	19	5.3	21	not performed due to i
5.0	8.0	1	not asked	24	6.1	18	11
5.0	8.0	1	not asked	6	5.9	25	9
5.0	8.0	1	not asked	2	6.3	24	11
6.0	8.0	1	not asked	2	6.9	25	12
7.0	8.0	1	not asked	23	5.8	18	10
8.0	8.0	1	not asked	7	6.9	missing	not performed due to i
8.0	8.0	3	not asked	8	6.7	28	12
8.0	8.0	1	not asked	20	7.0	12	not performed due to i
8.0	8.0	1	not asked	8	4.1	24	8
8.0	8.0	1	not asked	7	5.9	23	12
8.0	8.0	2	not asked	10	7.0	24	not performed due to i
9.0	8.0	1	not asked	7	6.0	26	13
10	8.0	1	not asked	9	5.9	27	17
10	8.0	1	not asked	25	6.1	19	10
10	8.5	1	not asked	21	6.5	missing	17
1.0	0	2	not asked	2	7.0	21	9
1.0	0	1	not asked	8	6.7	26	7
1.0	0	2	not asked	0	missing	22	9
1.5	0	1	not asked	7	6.0	21	11
1.5	0	3	not asked	10	5.4	27	12
2.0	0	1	not asked	18	missing	20	8
2.0	0	1	not asked	8	6.8	19	7
2.0	0	1	not asked	25	6.6	19	10
2.0	0	1	not asked	8	5.7	20	9
2.0	0	1	not asked	16	4.9	20	8
2.0	0	1	not asked	8	6.7	23	9
2.0	0	3	not asked	0	6.9	27	11
2.5	0	1	not asked	27	7.0	29	not performed due to i
2.5	0	1	not asked	14	7.0	22	not performed due to i
3.0	0	2	not asked	21	3.0	28	not performed due to i

For peer review only

3.0	0	2	not asked	1	7.0	23	11
3.0	0	1	not asked	4	6.4	23	not performed due to i
3.0	0	2	not asked	5	6.7	24	7
3.0	0	1	not asked	2	5.4	27	9
4.0	0	1	not asked	19	5.6	missing	12
4.0	0	3	not asked	6	5.9	24	13
4.0	0	1	not asked	13	6.7	27	11
5.0	0	1	not asked	2	6.5	25	14
5.0	0	1	not asked	10	7.0	16	not performed due to i
5.0	0	1	not asked	1	7.0	21	not performed due to i
5.0	0	1	not asked	7	6.0	18	10
5.0	0	1	not asked	17	4.8	11	not performed due to i
5.0	0	1	not asked	19	6.3	23	13
5.0	0	1	not asked	5	6.5	26	7
5.0	0	2	not asked	11	6.6	24	18
5.0	0	1	not asked	15	missing	20	9
5.5	0	1	not asked	0	missing	23	8
6.0	0	1	not asked	14	6.1	24	not performed due to i
6.5	0	1	not asked	12	4.0	24	10
7.0	0	1	not asked	13	3.5	missing	not performed due to i
8.0	0	1	not asked	3	6.9	22	27
8.0	0	3	not asked	1	7.0	19	7
8.0	0	1	not asked	5	6.1	26	not performed due to i
8.0	0	1	not asked	9	5.2	13	not performed due to i
8.0	0	3	not asked	16	6.8	19	not performed due to i
8.5	0	3	not asked	12	5.5	27	9
9.0	0	3	not asked	16	6.7	22	not performed due to i
9.0	0	1	not asked	21	6.4	20	not performed due to i
0	0	1	not asked	15	6.3	18	not performed due to i
0	0	1	not asked	10	missing	31	8
0	0	2	not asked	16	5.9	20	9
0	0	3	not asked	6	7.0	16	not performed due to i
0	0	1	not asked	18	4.1	22	16
0	0	3	not asked	4	6.4	24	5
0	0	1	not asked	2	5.3	17	not performed due to i
0	0	1	not asked	3	6.5	26	not performed due to i
0	0	2	not asked	14	6.9	29	not performed due to i
0	0	3	not asked	0	7.0	20	not performed due to i
0	0	1	not asked	18	5.5	24	12
0	0	1	not asked	5	missing	1	18
0	0	1	not asked	4	6.1	16	8
0	0	1	not asked	4	7.0	19	not performed due to i
0	0	1	not asked	7	7.0	18	11
0	0	1	not asked	4	4.9	missing	14
0	0	1	not asked	14	6.5	missing	not performed due to i
0	0	1	not asked	13	6.1	20	10
0	0	1	not asked	9	7.0	23	15
0	0	1	not asked	26	6.8	18	10
0	0	1	not asked	16	4.1	16	not performed due to i
0	0	1	not asked	2	6.6	17	15
0	0	1	not asked	8	6.9	18	12
0	0	1	not asked	14	missing	25	20

For peer review only

0	0	1	not asked	19	6.5	10	not performed due to i
0	0	2	not asked	6	6.3	25	10
0	0	2	not asked	9	5.0	26	9
0	0	2	not asked	12	5.7	20	10
0	0	2	not asked	9	6.9	28	26
0	0	3	not asked	13	6.6	9	not performed due to i
0	0	3	not asked	6	6.5	26	not performed due to i
0	0	3	not asked	14	4.3	16	not performed due to i
0	0	2	not asked	3	6.2	19	not performed due to i
0	0	1	not asked	5	missing	23	7
0	0	1	not asked	11	6.2	18	not performed due to i
0	0	1	not asked	9	6.9	21	12
0	0	1	not asked	7	6.5	22	9
0	0	1	not asked	14	5.9	26	10
0	0	1	not asked	11	6.5	26	10
0	0	1	not asked	10	5.5	25	not performed due to i
0	0	1	not asked	3	6.6	26	not performed due to i
0	0	1	not asked	1	6.6	25	8
0	0	1	not asked	7	5.5	23	10
0	0	2	not asked	7	5.1	27	not performed due to i
0	0	3	not asked	5	5.9	23	not performed due to i
0	0	3	not asked	19	5.7	29	8
0	0	3	not asked	8	7.0	21	not performed due to i
0	0	3	not asked	2	7.0	21	not performed due to i
0	0	3	not asked	6	5.9	25	not performed due to i
0	0	3	not asked	4	missing	missing	not performed due to i
0	0	1	not asked	2	6.6	22	15
0	0	1	not asked	10	3.6	12	18
0	0	1	not asked	3	3.5	0	10
0	0	1	not asked	19	6.7	25	7
0	0	2	not asked	15	4.8	25	10
0	0	2	not asked	5	6.3	17	not performed due to i
0	0	3	not asked	7	6.3	26	10
0	0	3	not asked	29	4.4	25	not performed due to i
7.0	10	1	not asked	18	6.3	28	8
9.0	10	1	not asked	31	3.5	24	12
10	10	1	not asked	15	6.6	21	15

For peer review only

increased fall risk or other medical reasons

increased fall risk or other medical reasons

increased fall risk or other medical reasons

increased fall risk or other medical reasons
increased fall risk or other medical reasons

increased fall risk or other medical reasons
increased fall risk or other medical reasons
increased fall risk or other medical reasons
increased fall risk or other medical reasons
increased fall risk or other medical reasons

increased fall risk or other medical reasons
increased fall risk or other medical reasons

increased fall risk or other medical reasons

increased fall risk or other medical reasons
increased fall risk or other medical reasons

increased fall risk or other medical reasons

increased fall risk or other medical reasons

increased fall risk or other medical reasons

increased fall risk or other medical reasons

increased fall risk or other medical reasons

increased fall risk or other medical reasons

For peer review only

increased fall risk or other medical reasons

increased fall risk or other medical reasons

increased fall risk or other medical reasons
increased fall risk or other medical reasons

increased fall risk or other medical reasons

increased fall risk or other medical reasons

increased fall risk or other medical reasons
increased fall risk or other medical reasons

For peer review only

increased fall risk or other medical reasons

increased fall risk or other medical reasons
increased fall risk or other medical reasons
increased fall risk or other medical reasons

increased fall risk or other medical reasons

increased fall risk or other medical reasons

increased fall risk or other medical reasons
increased fall risk or other medical reasons
increased fall risk or other medical reasons

increased fall risk or other medical reasons

increased fall risk or other medical reasons

For peer review only

increased fall risk or other medical reasons

increased fall risk or other medical reasons

increased fall risk or other medical reasons

increased fall risk or other medical reasons

increased fall risk or other medical reasons

increased fall risk or other medical reasons

increased fall risk or other medical reasons

increased fall risk or other medical reasons

increased fall risk or other medical reasons

increased fall risk or other medical reasons

increased fall risk or other medical reasons

increased fall risk or other medical reasons

increased fall risk or other medical reasons

increased fall risk or other medical reasons

increased fall risk or other medical reasons

increased fall risk or other medical reasons

increased fall risk or other medical reasons

For peer review only

increased fall risk or other medical reasons

increased fall risk or other medical reasons

increased fall risk or other medical reasons

increased fall risk or other medical reasons

increased fall risk or other medical reasons

increased fall risk or other medical reasons
increased fall risk or other medical reasons

increased fall risk or other medical reasons

increased fall risk or other medical reasons

increased fall risk or other medical reasons

increased fall risk or other medical reasons
increased fall risk or other medical reasons
increased fall risk or other medical reasons

increased fall risk or other medical reasons
increased fall risk or other medical reasons

increased fall risk or other medical reasons
increased fall risk or other medical reasons
increased fall risk or other medical reasons

For peer review only

increased fall risk or other medical reasons

increased fall risk or other medical reasons

increased fall risk or other medical reasons

increased fall risk or other medical reasons

increased fall risk or other medical reasons

increased fall risk or other medical reasons

increased fall risk or other medical reasons

increased fall risk or other medical reasons

increased fall risk or other medical reasons

increased fall risk or other medical reasons

increased fall risk or other medical reasons

increased fall risk or other medical reasons

increased fall risk or other medical reasons

increased fall risk or other medical reasons

For peer review only

increased fall risk or other medical reasons

increased fall risk or other medical reasons
increased fall risk or other medical reasons

increased fall risk or other medical reasons

increased fall risk or other medical reasons
increased fall risk or other medical reasons
increased fall risk or other medical reasons

increased fall risk or other medical reasons

increased fall risk or other medical reasons
increased fall risk or other medical reasons

increased fall risk or other medical reasons

increased fall risk or other medical reasons

increased fall risk or other medical reasons

increased fall risk or other medical reasons
increased fall risk or other medical reasons
increased fall risk or other medical reasons

For peer review only

increased fall risk or other medical reasons

increased fall risk or other medical reasons
increased fall risk or other medical reasons

increased fall risk or other medical reasons

increased fall risk or other medical reasons

increased fall risk or other medical reasons

increased fall risk or other medical reasons
increased fall risk or other medical reasons
increased fall risk or other medical reasons

increased fall risk or other medical reasons
increased fall risk or other medical reasons
increased fall risk or other medical reasons

increased fall risk or other medical reasons

increased fall risk or other medical reasons
increased fall risk or other medical reasons
increased fall risk or other medical reasons
increased fall risk or other medical reasons

increased fall risk or other medical reasons

increased fall risk or other medical reasons

increased fall risk or other medical reasons

For peer review only

increased fall risk or other medical reasons

increased fall risk or other medical reasons
increased fall risk or other medical reasons
increased fall risk or other medical reasons
increased fall risk or other medical reasons

increased fall risk or other medical reasons

increased fall risk or other medical reasons
increased fall risk or other medical reasons

increased fall risk or other medical reasons
increased fall risk or other medical reasons

increased fall risk or other medical reasons
increased fall risk or other medical reasons
increased fall risk or other medical reasons
increased fall risk or other medical reasons

increased fall risk or other medical reasons

increased fall risk or other medical reasons

For peer review only

STROBE Statement—Checklist of items that should be included in reports of ***cohort studies***

	Item No	Recommendation	Page No
**Title and abstract**	1	(*a*) Indicate the study's design with a commonly used term in the title or the abstract	1
		(*b*) Provide in the abstract an informative and balanced summary of what was done and what was found	2
**Introduction**			
Background/rationale	2	Explain the scientific background and rationale for the investigation being reported	4
Objectives	3	State specific objectives, including any prespecified hypotheses	5
**Methods**			
Study design	4	Present key elements of study design early in the paper	5
Setting	5	Describe the setting, locations, and relevant dates, including periods of recruitment, exposure, follow-up, and data collection	5
Participants	6	(*a*) Give the eligibility criteria, and the sources and methods of selection of participants. Describe methods of follow-up	5
		(*b*) For matched studies, give matching criteria and number of exposed and unexposed	
Variables	7	Clearly define all outcomes, exposures, predictors, potential confounders, and effect modifiers. Give diagnostic criteria, if applicable	5-6
Data sources/ measurement	8*	For each variable of interest, give sources of data and details of methods of assessment (measurement). Describe comparability of assessment methods if there is more than one group	5-6
Bias	9	Describe any efforts to address potential sources of bias	5-6
Study size	10	Explain how the study size was arrived at	5
Quantitative variables	11	Explain how quantitative variables were handled in the analyses. If applicable, describe which groupings were chosen and why	6-7
Statistical methods	12	(*a*) Describe all statistical methods, including those used to control for confounding	6-7
		(*b*) Describe any methods used to examine subgroups and interactions	6-7
		(*c*) Explain how missing data were addressed	6-7
		(*d*) If applicable, explain how loss to follow-up was addressed	n.a
		(*e*) Describe any sensitivity analyses	n.a.
**Results**			
Participants	13*	(a) Report numbers of individuals at each stage of study—eg numbers potentially eligible, examined for eligibility, confirmed eligible, included in the study, completing follow-up, and analysed	7
		(b) Give reasons for non-participation at each stage	n.a.
		(c) Consider use of a flow diagram	n.a.
Descriptive data	14*	(a) Give characteristics of study participants (eg demographic, clinical, social) and information on exposures and potential confounders	7
		(b) Indicate number of participants with missing data for each variable of interest	n.a.
		(c) Summarise follow-up time (eg, average and total amount)	5, 7
Outcome data	15*	Report numbers of outcome events or summary measures over time	

For peer review only - http://bmjopen.bmj.com/site/about/guidelines.xhtml

Main results	16	(*a*) Give unadjusted estimates and, if applicable, confounder-adjusted estimates and their precision (eg, 95% confidence interval). Make clear which confounders were adjusted for and why they were included<br>(*b*) Report category boundaries when continuous variables were categorized<br>(*c*) If relevant, consider translating estimates of relative risk into absolute risk for a meaningful time period	7
Other analyses	17	Report other analyses done—eg analyses of subgroups and interactions, and sensitivity analyses	7-8
**Discussion**			
Key results	18	Summarise key results with reference to study objectives	9
Limitations	19	Discuss limitations of the study, taking into account sources of potential bias or imprecision. Discuss both direction and magnitude of any potential bias	9-10
Interpretation	20	Give a cautious overall interpretation of results considering objectives, limitations, multiplicity of analyses, results from similar studies, and other relevant evidence	9
Generalisability	21	Discuss the generalisability (external validity) of the study results	10
**Other information**			
Funding	22	Give the source of funding and the role of the funders for the present study and, if applicable, for the original study on which the present article is based	11

*Give information separately for exposed and unexposed groups.

Note: An Explanation and Elaboration article discusses each checklist item and gives methodological background and published examples of transparent reporting. The STROBE checklist is best used in conjunction with this article (freely available on the Web sites of PLoS Medicine at http://www.plosmedicine.org/, Annals of Internal Medicine at http://www.annals.org/, and Epidemiology at http://www.epidem.com/). Information on the STROBE Initiative is available at http://www.strobe-statement.org.

For peer review only

BMJ Open

Changes of Perceptions and Behaviours during the Phases of COVID-19 Pandemic in German Elderly People with Neurological Disorders: An Observational Study using Telephone Interviews

Journal:	*BMJ Open*
Manuscript ID	bmjopen-2020-045780.R1
Article Type:	Original research
Date Submitted by the Author:	10-Dec-2020
Complete List of Authors:	Zipprich, Hannah; Jena University Hospital, Department of Neurology<br>Schönenberg, Aline; Jena University Hospital, Neurology<br>Teschner, Ulrike; Jena University Hospital, Neurology<br>Prell, Tino; Jena University Hospital, Neurology
**Primary Subject Heading**:	Geriatric medicine
Secondary Subject Heading:	Health services research, Neurology
Keywords:	Neurology < INTERNAL MEDICINE, COVID-19, Anxiety disorders < PSYCHIATRY

SCHOLARONE™
Manuscripts

I, the Submitting Author has the right to grant and does grant on behalf of all authors of the Work (as defined in the below author licence), an exclusive licence and/or a non-exclusive licence for contributions from authors who are: i) UK Crown employees; ii) where BMJ has agreed a CC-BY licence shall apply, and/or iii) in accordance with the terms applicable for US Federal Government officers or employees acting as part of their official duties; on a worldwide, perpetual, irrevocable, royalty-free basis to BMJ Publishing Group Ltd ("BMJ") its licensees and where the relevant Journal is co-owned by BMJ to the co-owners of the Journal, to publish the Work in this journal and any other BMJ products and to exploit all rights, as set out in our licence.

The Submitting Author accepts and understands that any supply made under these terms is made by BMJ to the Submitting Author unless you are acting as an employee on behalf of your employer or a postgraduate student of an affiliated institution which is paying any applicable article publishing charge ("APC") for Open Access articles. Where the Submitting Author wishes to make the Work available on an Open Access basis (and intends to pay the relevant APC), the terms of reuse of such Open Access shall be governed by a Creative Commons licence – details of these licences and which Creative Commons licence will apply to this Work are set out in our licence referred to above.

Other than as permitted in any relevant BMJ Author's Self Archiving Policies, I confirm this Work has not been accepted for publication elsewhere, is not being considered for publication elsewhere and does not duplicate material already published. I confirm all authors consent to publication of this Work and authorise the granting of this licence.

For peer review only

BMJ open

Changes of Perceptions and Behaviours during the Phases of COVID-19 Pandemic in German Elderly People with Neurological Disorders: An Observational Study using Telephone Interviews

Hannah M. Zipprich, MD[1,2], Aline Schönenberg[1], Ulrike Teschner, PhD[1] and Tino Prell, MD[1,2,*]

[1] Department of Neurology, Jena University Hospital, Jena, Germany
[2] Center for Healthy Ageing, Jena University Hospital, Jena, Germany

* Correspondence: Tino.prell@med.uni-jena.de; Tel.: +49 3641 9323497

Word count (excl. title page, abstract, references, figures and tables): 2390 words

For peer review only

ABSTRACT

Objectives: Describing perceived limitations in everyday life, psychological burden and approval to easing of measures during the COVID-19 phases in elderly people with neurological disorders.

Design: Observational, prospective study

Setting: This is a monocentric study conducted at a university hospital in Germany.

Participants: Overall, 452 elderly people participated in the NeuroGerAdh study (DRKS00016774) and were interviewed by telephone between March 18 and August 30, 2020.

Results: Overall, 307 (67.9%) patients had relevant limitations in daily life due to the measures. These limitations significantly decreased during the pandemic phases. At the beginning of the pandemic, people complained about restricted social contacts and mobility, which were the most common reasons for perceived limitations in daily life. Later, since June 2020, wearing a mouth–nose mask had become the main reason for perceived limitations. In the elastic net regularisation model higher perceived limitations in daily life were among others associated with younger age and earlier pandemic phases. Higher psychological burden was mainly associated with early pandemic phase, younger age, and depression.The perceived psychological burden decreased as the pandemic phases passed, even though the reasons for psychological burden (anxiety or fear of infection, insecurity and concerns) did not remarkably change during the phases. From June 16, 2020, the patients were asked whether they approve the easing of measures. Sixty-seven of 136 patients (49.3%) approved and 55 (40.4%) did not. The common reasons for disapproval were fear of increased risk of infection and irresponsible behaviour of other people.

Conclusion: While limitations in daily life decreased during the study period, anxiety remains a common psychological burden in elderly sick people, and this needs special attention. Accordingly, most people do not approve easing of measures. Special strategies are needed to cope with changing measures during the COVID-19 pandemic.

KEYWORDS: neurogeriatric; COVID-19; anxiety; easing of measures

For peer review only

ARTICLE SUMMARY

Strengths and limitations of this study

- The inclusion of underrepresented elderly sick people.
- The availability of clinical data for depression, cognitive ability and mobility for the telephone-interviewed participants.
- The monocentric study design and focus on people with neurological disorders.
- The cross-sectional design does not allow statements about causality.

For peer review only

INTRODUCTION

Severe acute respiratory syndrome coronavirus 2 (SARS-CoV-2), a novel virus causing coronavirus disease 2019 (COVID-19) infection, has resulted in a pandemic. Local and national governments have taken unprecedented measures in response to the outbreak of SARS-CoV-2-induced COVID-19, including the isolation of patients, enforcement of quarantine of all contacts, cancellation of public transportation, exit controls, travel restrictions, social contact restrictions and requirement of people to wear mouth–nose masks.[1 2]

Considering the globally and locally rising infection rates, the German federal state issued different ordinances to contain the spread of the virus. The first ordinance published in March 2020 included a lockdown with limited social contacts, maintaining a minimum distance of at least 1.5m in public and closing of service establishments and restaurants as well as teaching facilities. A maximum of two people from different households were allowed to meet (see also Thüringer Verordnung über erforderliche Maßnahmen zur Eindämmung der Ausbreitung des Coronavirus SARS CoV 2, 2020). Since April 2020, governments have been requiring people to wear mouth–nose masks in shops and public transportation. In May 2020, Germany announced relevant easing of measures against the coronavirus pandemic: resuming regular operating hours of kindergartens and schools, reopening of restaurants and allowing meetings of up to five people from different households. Since the beginning of June 2020, further easing of measures has been occurring. For example, visits to nursing homes and larger family celebrations are possible again. Thuringia, in particular, was the first federal state to lift the contact restrictions. The new standard regulations deployed since June 2020 still require people to practice social distancing and wear mouth–nose masks in shops and public transportation, but contain only recommendations and no rules for restricting social contacts (see also Thüringer Verordnung zur Neuordnung der erforderlichen Maßnahmen zur Eindämmung der Ausbreitung des Coronavirus SARS-CoV-2, 2020).

It is understandable that such drastic measures have a considerable impact on the physical and psychological well-being of the population.[3-5] In particular, higher levels of depression, stress, and anxiety were found during COVID-19 pandemic.[5-7] Especially, the elderly population, being particularly vulnerable to severe cases of COVID-19, is at risk of increased

For peer review only

anxiety and depression.[8] So far, however, the effect of the measures in the older population and changes in perceptions when measures were being relaxed have not been sufficiently investigated. Furthermore, little is known about how the measures are perceived by older people with chronic diseases or relevant functional impairments. In our opinion, the results of surveys of younger or community-dwelling elderly people, e.g. COVID-19 Snapshot Monitoring (COSMO) [9]), are not necessarily transferable to this cohort. Therefore, we interviewed elderly people who were previously hospitalized in our hospital because of a neurological disorder. We were particularly interested in how the perception of the measures changes over time. Therefore, we explored the impact of the measures during the three phases of the coronavirus pandemic in Germany: Phase 1 (March 18, 2020–May 15, 2020) with the lockdown, Phase 2 (May 16, 2020–June 05, 2020) with the first easing of measures and Phase 3 (since June 06, 2020) with further easing of measures.

METHODS

Study design and assessments

The people who were enrolled in the NeuroGerAdh study (DRKS00016774) were interviewed via telephone between March 18, 2020 and August 30, 2020. The NeuroGerAdh study is a longitudinal observational study of predictors of nonadherence to medication in patients with neurological disorders.[10] Here, elderly people who were treated at the Department of Neurology at Jena University Hospital were consecutively recruited and assessed using comprehensive geriatric assessment and an adherence questionnaire and two follow-up telephone calls to explore changes in medication after discharge from hospital. The study was approved by the local ethics committee (approval number 5290-10/17) of Jena University Hospital. All patients provided written informed consent. During the COVID-19 pandemic, the following questions were added to the interview:

1. Are or were you infected with coronavirus? Yes/no (if yes, when?)
2. Are or have you already been quarantined? Yes/no (if yes, how long?)
3. For which activities do you still leave the house/apartment?
    - Shopping
    - Visiting to the doctor
    - Walking/hiking/cycling

For peer review only

- Working in the garden
- Doing sports
- Visiting family
- Meeting with friends
- Not at all, I am being cared for

4. Please indicate on a scale of 0–10 how much you are limited in daily life by the measures against COVID-19 (0 being 'no limitation of my daily life' and 10 being 'strongest restrictions of my daily life'). If >0, please specify what you are limited by/what you feel is a limitation.

5. Please indicate on a scale of 0–10 how much of a psychological burden the restrictions put on you (0 being 'not burdening at all' and 10 being 'very strongly burdening').

6. Since June 16, 202,0 we additionally asked, 'Do you approve the easing of the measures?' Yes/no (if no, why?)

7. From June 16, 2020, the people were also asked, 'Do you approve the easing of the measures?' Yes/no (if no, why?)

Answers to questions 4–7 were evaluated qualitatively and categorised into specific subject areas. In addition, the following data were used for analyses: age, gender, main neurological disorder, marital status (single, divorced/widowed or married), level of education (high: German *Abitur* or university; medium: German *Realschule* or the General Certificate of Secondary Education; low: German *Hauptschule* or no school), cognitive function (Montreal Cognitive Assessment (MoCA)), depressive mood (Beck Depression Inventory II (BDI-II)), patient–physician relationship (Health Care Climate Questionnaire (HCCQ)) and Timed 'Up & Go' (TUG) test in seconds to assess a person's mobility and risk of falling.

Patient and Public Involvement statement

Patients or the public were not involved in the design, or conduct, or reporting, or dissemination plans of our research.

For peer review only

Statistical analysis

Statistical analysis was performed using Statistical Package for the Social Sciences (version 25.0; IBM Corp., Armonk, NY, USA) and R 3.6.2 (R Foundation for Statistical Computing, Vienna, Austria). The data were analysed using descriptive statistics: means, standard deviations, frequencies and percentages. No techniques were used to replace missing data. Comparisons of clinical parameters between patients from the three phases were performed using the Kruskal–Wallis and chi-square tests. Elastic net regularization was used to study the association between perceived limitations in daily life and perceived psychological burden with the following independent variables: age, gender, marital state (married or unmarried), education level (high, middle and low), pandemic phase, BDI, HCCQ, MoCA and TUG in seconds. Generally, elastic net regularisation leads to parsimonious models, which are easier to interpret [11]. Variable selection is performed by shrinking parameters towards zero and attenuating overfitting, a well-known problem if regression models are applied with a large number of predictors. Ten-fold cross validation was applied to choose the best model with the lowest mean cross-validated error. Within the elastic net algorithm, variables remain in the model if the prediction error averaged over the ten cross-validation samples is reduced. In contrast to ordinary least squares regression or LASSO regularisation, the elastic net algorithm performs well in highly correlated variables, either including all of them with similar regression coefficients or excluding all of them from the best model. Regressions coefficients and p-values of the model were reported. Elastic net regularization was performed with the package glmnet in R 3.6.2.

For peer review only

RESULTS

Among the 452 people were interviewed in this study (Table 1), 230 (50.9%) were interviewed in Phase 1, 41 (9.1%) in Phase 2 and 181 (40.0%) in Phase 3. Only two people (0.4%) had COVID-19 infection and 18 people (4%) had been in quarantine before the interview. The people who were interviewed in the three phases did not differ in terms of age (p = 0.68), BDI (p = 0.16), HCCQ (p = 0.76), MoCa (p = 0.49), TUG (p = 0.38), gender (p = 0.53), marital state (p = 0.58) and education level (p = 0.69).

Table 1. Characteristics of the entire cohort

		m	SD
Age		69.5	8.6
Beck Depression Inventory II		9.7	7.4
Health Care Climate Questionnaire		5.9	1.1
Montreal Cognitive Assessment		22.8	4.7
Timed Up & Go		10.6	4.5
		**n**	**%**
Sex	Female	205	45.4
	Male	247	54.6
Marital status	Single	131	29.5
	Married	313	70.5
Education level	High	153	34.5
	Middle	156	35.2
	Low	134	30.2
Diagnosis group	Movement disorders	125	27.7
	Cerebrovascular disorders	116	25.7
	Epilepsy	24	5.3
	Neuromuscular disorders	105	23.2
	Others	82	18.1

Overall, 307 (67.9%) people reported having relevant limitations in their daily routine. The perceived limitations in daily life caused by the measures decreased significantly during the pandemic phases (Figure 1). The slight increase in perceived limitations from Phase 2 to Phase 3 was not significant. The reasons for the perceived limitations changed during the pandemic phases. At the beginning of the pandemic, people considered restricted social contacts and mobility as the most common reasons for perceived limitations in daily life. Later, at Phase 3, wearing mouth–nose masks had become the main perceived limitation in daily life (Figure 1). Corresponding to the easing of measures, social activities (meeting family members and friends) and visits to doctors increased from Phase 1 to Phase 3 (Figure 2).

In the elastic net regularisation model higher perceived limitations in daily life were significantly associated with younger age and earlier pandemic phase (Table 2). To a lesser extent, also other factors determined how strongly the limitations were felt in daily life. The restrictions were perceived to be less drastic in daily life for married people, people with lower levels of education, and people with a poorer physician-patient relationship (lower HCCQ). In contrast, the limitations in daily life were perceived as more severe for men,

For peer review only

people with a high level of education or higher MoCa, people with depression (BDI) and people with mobility impairments (higher TUG) (Table 2).

Table 2. Predictors of perceived limitations in daily living

	Coefficient	p-value
Age *	-0.11	<0.001
Pandemic phase II *	-2.93	<0.001
Pandemic phase III *	-2.20	<0.001
Marital status (married)	-0.68	0.11
Education (middle)	-0.80	0.08
Education (low)	-0.49	0.31
Health Care Climate Questionnaire	-0.13	0.47
Male gender	0.12	0.76
Beck Depression Inventory II	0.01	0.73
Montreal Cognitive Assessment	0.07	0.18
Timed Up & Go	0.03	0.47
$\chi^2(11)$ = 534.74, p = 0.00, Pseudo-R^2 (Cragg-Uhler) = 0.23, AIC = 1175.04, BIC = 1220.12.* significant predictors.		

Furthermore, the perceived psychological burden decreased during the pandemic phases (Figure 3). Reasons, such as fear of infection, worries on how it should go on (insecurity and concerns) and reduced social contacts, for the psychological burden did not remarkably change during the phases (Figure 3). In the elastic net regularisation model higher psychological burden was significantly associated with early pandemic phase, lower age, and higher BDI. In addition perceived psychological burden was higher in women, people with high education level or higher MoCa, people with mobility impairments, single living people, and people with poorer physician-patient relationship (lower HCCQ) (Table 3).

Table 3. Predictors of perceived psychological burden

	Coefficient	p-value
Age *	-0.06	0.01
Pandemic phase II *	-2.62	<0.001
Pandemic phase III *	-2.07	<0.001
Marital status (married)	-0.63	0.12
Education (middle)	-0.64	0.14
Education (low)	-0.54	0.25
Health Care Climate Questionnaire	-0.25	0.15
Male gender	-0.55	0.13
Beck Depression Inventory II *	0.06	0.01
Montreal Cognitive Assessment	0.08	0.09
Timed Up & Go	0.02	0.62
$\chi^2(11)$ = 599.29, p = 0.00, Pseudo-R^2 (Cragg-Uhler) = 0.23,AIC = 1301.61, BIC = 1348.00, * significant predictors.		

From June 16, 2020, the people were also asked if they approve the easing of measures. Among the 136 interviewed patients, 67 (49.3%) approved, 55 (40.4) did not approve and 14 could not make a decision. The common reasons not to approve the easing of measures were fear of increased risk of infection and irresponsible behaviour of other people. Patients who did or did not approve easing of measures did not differ in terms of age (p = 0.85), gender (p = 0.31), education level (p = 0.80), marital state (p = 0.52), BDI (p = 0.50), TUG time (p = 0.27), MoCa (p = 0.31) and patient–physician relationship according to the HCCQ (p = 0.44). Both groups also did not differ in the perceived limitations in daily life (p = 0.38) and psychological burden (p = 0.51) caused by the measures.

DISCUSSION

Our study shows how perceived limitations of daily life declined with ongoing easing of measures and the reasons for this change during different pandemic phases. Perceived limitations due to the measures were higher at the beginning of the pandemic and in younger aged persons. In our models early pandemic phase and younger age were significant predictors of perceived limitations in daily life. There may be several reasons why older people perceived the limitations as less severe. For example, fewer responsibilities and more experiences with previous threatening situations can additionally explain the lower levels of perceived limitations among the elderly.[12] To a lesser extent, factors other than the pandemic phase and age also played a role in how drastic the measures were experienced by the respondents. Independent of age and pandemic phase, the measures were particularly drastic in terms of daily life for men, educated people, people with depression and people with mobility impairments.

The most important limitation was the social contact restrictions. Even after various easing of the measures in Phase 3, these were still the main reasons for perceived limitations in everyday life. Although perceived limitations were associated with younger age, the elderly people are particularly susceptible to the effects of uncertainty and lack of social contacts.[13] After the easing of the measures, the most frequent reason for perceived limitations in everyday life was the use of the mouth–nose mask (46%). This is in line with a recent cross-sectional Brazilian study of 1277 participants. Here, most respondents (67.3%) reported that

For peer review only

the use of masks bothers them in some way (feel trapped, suffocated, feel shortness of breath, feel discomfort in the ears due to the elastics, fogs up the glasses)[14]. Overall, even though people reported discontent over the mandatory use of masks, the general population followed the rule relatively well, as wearing masks signals empathy and prosocial behaviour toward members of the risk group.[15] Parallel to the easing of the measures, social activities, such as visiting friends also increased.

On the psychological burden side, a relevant fear of coronavirus infection is indicated throughout all pandemic phases. This fits the results of a representative survey of people in Wuhan (the epicentre of the COVID-19 outbreak in China) and Shanghai in February 2020. Here, the prevalence of moderate or severe anxiety was significantly higher in Wuhan than Shanghai. Anxiety was predicted with perceived harm of the disease and confusion about information reliability.[16] This fear increased with the significant easing of measures in Phase 3. It is important to note here that the respondents were elderlies with various neurological and internal diseases. Many respondents stated that they were at increased risk of a severe course of COVID-19 disease and thus belonged to the risk group. This may explain the high level of anxiety.[8] Appropriately, approximately half of the respondents were critical on the easing of measures. However, the feeling of insecurity and worries about the future decreased over the course of the pandemic. This is the best indication that the respondents adapted to the specific requirements of the situation and were better informed than at the beginning of the pandemic, since sufficient information leads to less psychological burden like anxiety.[17] In the elastic net regularisation model the higher psychological burden was mainly associated with early pandemic phase, younger age, and depression according to the BDI. While limitations in daily life were more relevant for men, higher psychological burden was associated with female gender. Moreover, high educated people, people with mobility impairments, and single living people claimed to be severely psychologically burdened by the measures.

The study has some limitations. It is important to emphasise that the data are cross-sectional, that is, different people were interviewed in the respective phases. Although the demographic and clinical parameters are comparable between the interviewed patients in the different phases, which is the usual design for other regular surveys in the context of the COVID-19 pandemic (e.g. COVID-19 Snapshot Monitoring (COSMO)[9]), distortions caused by

For peer review only

this design cannot be excluded. Furthermore, the survey focused on elderly and sick people. Although this limits the generalizability of the results, these data are important because older people are often underrepresented in other surveys (e.g. COSMO).

For peer review only

FOOTNOTES

Acknowledgment: We thank Marieke Jäger and Verena Buchholz for assistance in data acquisition.

Author Contributions: AS, UT, HZ collected data, performed the statistical analysis and drafted the initial manuscript. TP provided the concept and design of the study and was involved in the analysis. HZ was involved in the interpretation of the data. TP revised the manuscript for important intellectual content. All authors read and approved the final manuscript.

Competing interests: None declared.

Ethics approval: All procedures performed in studies involving human participants were in accordance with the ethical standards of the institutional research committee (ethics committee of the Jena University Hospital, 4572-10/15) and with the 1964 Helsinki declaration and its later amendments or comparable ethical standards.

Funding: This work was supported by a Bundesministerium für Bildung und Forschung grant to Tino Prell (01GY1804). This funding source had no role in the design of this study and will not have any role during its execution, analyses, interpretation of the data, or decision to submit results.

Data sharing statement: Dataset is available upon reasonable request from the corresponding author for scientific purpose. .

For peer review only

REFERENCES

1. Koo JR, Cook AR, Park M, et al. Interventions to mitigate early spread of SARS-CoV-2 in Singapore: a modelling study. *Lancet Infect Dis* 2020 doi: 10.1016/S1473-3099(20)30162-6
2. Prem K, Liu Y, Russell TW, et al. The effect of control strategies to reduce social mixing on outcomes of the COVID-19 epidemic in Wuhan, China: a modelling study. *Lancet Public Health* 2020 doi: 10.1016/S2468-2667(20)30073-6
3. Torales J, O'Higgins M, Castaldelli-Maia JM, et al. The outbreak of COVID-19 coronavirus and its impact on global mental health. 2020;66(4):317-20. doi: 10.1177/0020764020915212
4. Zipprich HM, Teschner U, Witte OW, et al. Knowledge, Attitudes, Practices, and Burden During the COVID-19 Pandemic in People with Parkinson's Disease in Germany. *J Clin Med* 2020;9(6) doi: 10.3390/jcm9061643
5. Kecojevic A, Basch CH, Sullivan M, et al. The impact of the COVID-19 epidemic on mental health of undergraduate students in New Jersey, cross-sectional study. *PLoS One* 2020;15(9):e0239696. doi: 10.1371/journal.pone.0239696 [published Online First: 2020/10/01]
6. Wang X, Hegde S, Son C, et al. Investigating Mental Health of US College Students During the COVID-19 Pandemic: Cross-Sectional Survey Study. *Journal of medical Internet research* 2020;22(9):e22817. doi: 10.2196/22817 [published Online First: 2020/09/09]
7. Wang PW, Ko NY, Chang YP, et al. Subjective Deterioration of Physical and Psychological Health during the COVID-19 Pandemic in Taiwan: Their Association with the Adoption of Protective Behaviors and Mental Health Problems. *International journal of environmental research and public health* 2020;17(18) doi: 10.3390/ijerph17186827 [published Online First: 2020/09/24]
8. Dubey S, Biswas P, Ghosh R, et al. Psychosocial impact of COVID-19. *Diabetes & metabolic syndrome* 2020;14(5):779-88. doi: 10.1016/j.dsx.2020.05.035 [published Online First: 2020/06/12]
9. Betsch C, Wieler L, Bosnjak M, et al. COVID-19 Snapshot Monitoring (COSMO): monitoring knowledge, risk perceptions, preventive behaviours, and public trust in the current coronavirus outbreak. *Psych Archives* 2020 doi: http://dx.doi.org/10.23668/psycharchives.2776
10. Prell T. Adherence to medication in neurogeriatric patients: an observational cross-sectional study. *BMC Public Health* 2019;19(1):1012. doi: 10.1186/s12889-019-7353-5
11. Zou H, Hastie T. Regularization and variable selection via the elastic net. *Journal Of The Royal Statistical Society* 2005;67(2):19. doi: 10.1111/j.1467-9868.2005.00503.x
12. Ngo EB. When Disasters and Age Collide: Reviewing Vulnerability of the Elderly. 2001;2(2):80-89. doi: doi:10.1061/(ASCE)1527-6988(2001)2:2(80)
13. Glowacz F, Schmits E. Psychological distress during the COVID-19 lockdown: The young adults most at risk. *Psychiatry Research* 2020;293:113486. doi: 10.1016/j.psychres.2020.113486 [published Online First: 2020/10/03]
14. Cotrin P, Bahls AC, da Silva DdO, et al. The Use of Facemasks During the COVID-19 Pandemic by the Brazilian Population. *J Multidiscip Healthc* 2020;13:1169-78. doi: 10.2147/JMDH.S281524
15. Betsch C, Korn L, Sprengholz P, et al. Social and behavioral consequences of mask policies during the COVID-19 pandemic. *Proceedings of the National Academy of Sciences of the United States of America* 2020;117(36):21851-53. doi: 10.1073/pnas.2011674117 [published Online First: 2020/08/21]
16. Qian M, Wu Q, Wu P, et al. Anxiety levels, precautionary behaviours and public perceptions during the early phase of the COVID-19 outbreak in China: a population-based cross-sectional survey. *BMJ open* 2020;10(10):e040910-e10. doi: 10.1136/bmjopen-2020-040910
17. Rodríguez-Rey R, Garrido-Hernansaiz H, Collado S. Psychological Impact and Associated Factors During the Initial Stage of the Coronavirus (COVID-19) Pandemic Among the General Population in Spain. 2020;11(1540) doi: 10.3389/fpsyg.2020.01540

For peer review only

FIGURE LEGENDS

Figure 1. Change and reasons of perceived limitations in daily life during the COVID-19 pandemic.

0 = 'no limitation of my daily life' to 10 = 'strongest restrictions of my daily life'

Figure 2. Percentage of people performing different activities in different COVID-19 pandemic phases.

Figure 3. Change and reasons of psychological burden during the COVID-19 pandemic.

0 = 'not burdening at all' to 10 = 'very strongly burdening'

For peer review only

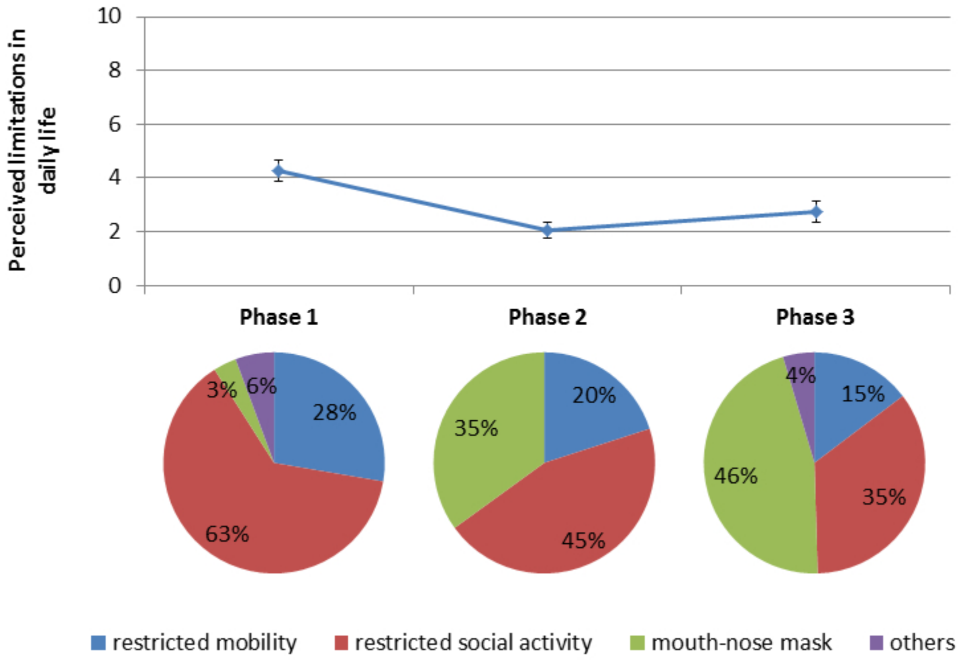

Figure 1. Change and reasons of perceived limitations in daily life during the COVID-19 pandemic.

63x44mm (300 x 300 DPI)

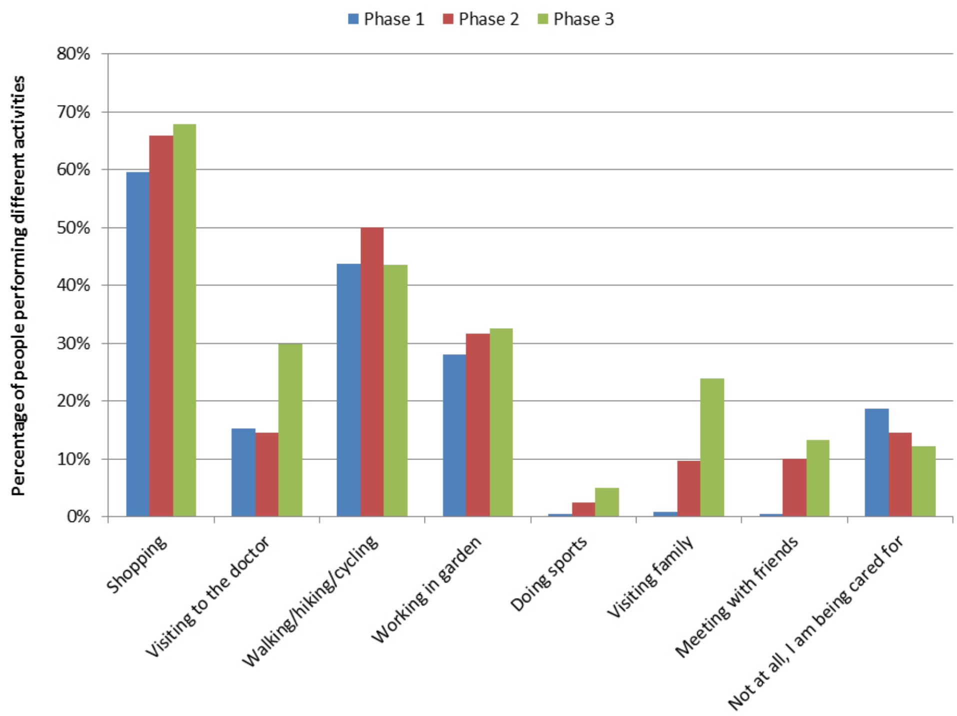

Figure 2. Percentage of people performing different activities in different COVID-19 pandemic phases.

81x60mm (300 x 300 DPI)

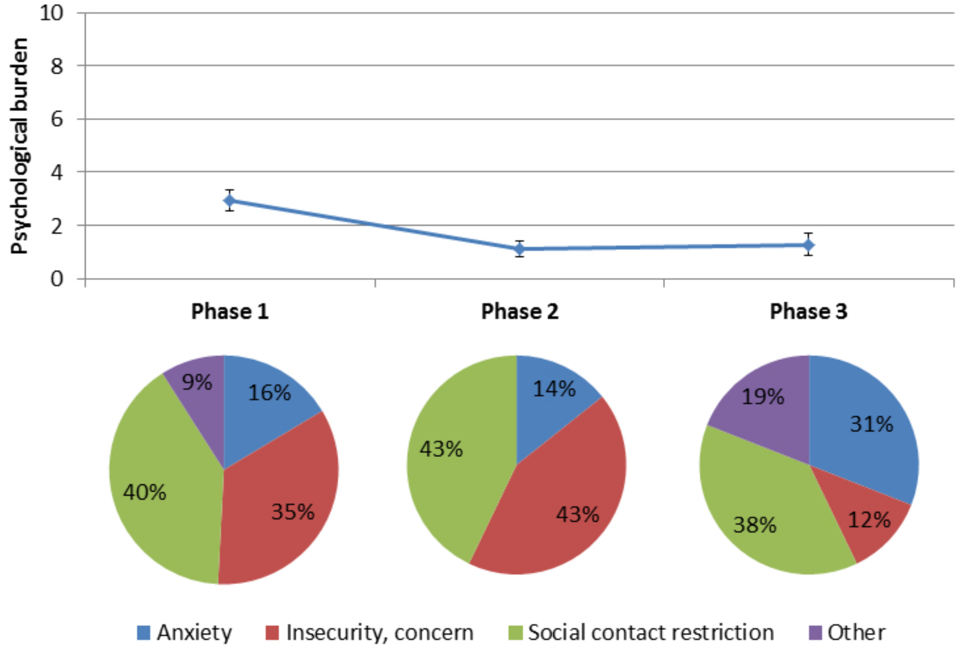

Figure 3. Change and reasons of psychological burden during the COVID-19 pandemic.

64x44mm (300 x 300 DPI)

STROBE Statement—Checklist of items that should be included in reports of ***cohort studies***

	Item No	Recommendation	Page No
**Title and abstract**	1	(*a*) Indicate the study's design with a commonly used term in the title or the abstract	1
		(*b*) Provide in the abstract an informative and balanced summary of what was done and what was found	2
**Introduction**			
Background/rationale	2	Explain the scientific background and rationale for the investigation being reported	4
Objectives	3	State specific objectives, including any prespecified hypotheses	5
**Methods**			
Study design	4	Present key elements of study design early in the paper	5
Setting	5	Describe the setting, locations, and relevant dates, including periods of recruitment, exposure, follow-up, and data collection	5
Participants	6	(*a*) Give the eligibility criteria, and the sources and methods of selection of participants. Describe methods of follow-up	5
		(*b*) For matched studies, give matching criteria and number of exposed and unexposed	
Variables	7	Clearly define all outcomes, exposures, predictors, potential confounders, and effect modifiers. Give diagnostic criteria, if applicable	5-6
Data sources/ measurement	8*	For each variable of interest, give sources of data and details of methods of assessment (measurement). Describe comparability of assessment methods if there is more than one group	5-6
Bias	9	Describe any efforts to address potential sources of bias	5-6
Study size	10	Explain how the study size was arrived at	5
Quantitative variables	11	Explain how quantitative variables were handled in the analyses. If applicable, describe which groupings were chosen and why	6-7
Statistical methods	12	(*a*) Describe all statistical methods, including those used to control for confounding	6-7
		(*b*) Describe any methods used to examine subgroups and interactions	6-7
		(*c*) Explain how missing data were addressed	6-7
		(*d*) If applicable, explain how loss to follow-up was addressed	n.a
		(*e*) Describe any sensitivity analyses	n.a.
**Results**			
Participants	13*	(a) Report numbers of individuals at each stage of study—eg numbers potentially eligible, examined for eligibility, confirmed eligible, included in the study, completing follow-up, and analysed	7
		(b) Give reasons for non-participation at each stage	n.a.
		(c) Consider use of a flow diagram	n.a.
Descriptive data	14*	(a) Give characteristics of study participants (eg demographic, clinical, social) and information on exposures and potential confounders	7
		(b) Indicate number of participants with missing data for each variable of interest	n.a.
		(c) Summarise follow-up time (eg, average and total amount)	5, 7
Outcome data	15*	Report numbers of outcome events or summary measures over time	

For peer review only

Main results	16	(*a*) Give unadjusted estimates and, if applicable, confounder-adjusted estimates and their precision (eg, 95% confidence interval). Make clear which confounders were adjusted for and why they were included<br>(*b*) Report category boundaries when continuous variables were categorized<br>(*c*) If relevant, consider translating estimates of relative risk into absolute risk for a meaningful time period	7
Other analyses	17	Report other analyses done—eg analyses of subgroups and interactions, and sensitivity analyses	7-8
**Discussion**			
Key results	18	Summarise key results with reference to study objectives	9
Limitations	19	Discuss limitations of the study, taking into account sources of potential bias or imprecision. Discuss both direction and magnitude of any potential bias	9-10
Interpretation	20	Give a cautious overall interpretation of results considering objectives, limitations, multiplicity of analyses, results from similar studies, and other relevant evidence	9
Generalisability	21	Discuss the generalisability (external validity) of the study results	10
**Other information**			
Funding	22	Give the source of funding and the role of the funders for the present study and, if applicable, for the original study on which the present article is based	11

*Give information separately for exposed and unexposed groups.

Note: An Explanation and Elaboration article discusses each checklist item and gives methodological background and published examples of transparent reporting. The STROBE checklist is best used in conjunction with this article (freely available on the Web sites of PLoS Medicine at http://www.plosmedicine.org/, Annals of Internal Medicine at http://www.annals.org/, and Epidemiology at http://www.epidem.com/). Information on the STROBE Initiative is available at http://www.strobe-statement.org.

For peer review only
